# RNA nanotherapeutics for hepatocellular carcinoma treatment

**DOI:** 10.7150/thno.102964

**Published:** 2025-01-01

**Authors:** Yihang Yuan, Weijie Sun, Jiaqi Xie, Ziheng Zhang, Jing Luo, Xiangfei Han, Yongfu Xiong, Yang Yang, Yang Zhang

**Affiliations:** 1 The Comprehensive Cancer Centre of Nanjing Drum Tower Hospital, Affiliated Hospital of Medical School, Nanjing University and Clinical Cancer Institute of Nanjing University, Nanjing 210008, China.; 2 Department of Hepatobiliary Surgery, Academician (Expert) Workstation, Affiliated Hospital of North Sichuan Medical College, Nanchong 637600, China.; 3 School of Life Sciences, Jiangsu University, Zhenjiang 212013, China.; 4 Department of Medical Oncology, The First Affiliated Hospital of Bengbu Medical University, Bengbu 233004, China.; 5 Department of General Surgery Nanjing Drum Tower Hospital, Affiliated Hospital of Medical School Nanjing University, Nanjing 210008, China.; 6 Center for Nanomedicine and Department of Anesthesiology, Perioperative and Pain Medicine, Brigham and Women's Hospital, Harvard Medical School, Boston, MA 02115, USA.; 7 Department of Urology, Brigham and Women's Hospital, Harvard Medical School, Boston, MA 02115, USA.

**Keywords:** RNA nanotherapeutics, Gene regulation, Protein restoration, Gene activation, Hepatocellular carcinoma

## Abstract

Hepatocellular carcinoma (HCC) remains a leading cause of cancer-related mortality worldwide, particularly due to the limited effectiveness of current therapeutic options for advanced-stage disease. The efficacy of traditional treatments is often compromised by the intricate liver microenvironment and the inherent heterogeneity. RNA-based therapeutics offer a promising alternative, utilizing the innovative approach of targeting aberrant molecular pathways and modulating the tumor microenvironment. The integration of nanotechnology in this field, through the development of advanced nanocarrier delivery systems, especially lipid nanoparticles (LNPs), polymer nanoparticles (PNPs), and bioinspired vectors, enhances the precision and efficacy of RNA therapies. This review highlights the significant progress in RNA nanotherapeutics for HCC treatment, covering micro RNA (miRNA), small interfering RNA (siRNA), message RNA (mRNA), and small activating RNA (saRNA) mediated gene silencing, therapeutic protein restoration, gene activation, cancer vaccines, and concurrent therapy. It further comprehensively discusses the prevailing challenges within this therapeutic landscape and provides a forward-looking perspective on the potential of RNA nanotherapeutics to transform HCC treatment.

## Introduction

Hepatocellular carcinoma (HCC) is the most common primary liver malignancy and a leading cause of cancer-related deaths worldwide, with a five-year survival rate of approximately 18% due to asymptomatic progression and late detection 1-3. Current treatments for HCC, including surgical resection, liver transplantation, local ablative therapies, and systemic treatments such as chemotherapy and targeted therapy, are often ineffective, especially at advanced stages 4-7. Fewer than 20% of patients are eligible for surgery, systemic chemotherapy offers limited benefits with significant toxicity, and targeted agents provide only modest survival improvements, frequently hindered by drug resistance and adverse effects. Additionally, tumor heterogeneity and the unique liver microenvironment promote drug resistance and reduce therapeutic efficacy by shielding cancer cells and rapidly metabolizing drugs 8-11. These issues highlight the urgent need for novel therapeutic strategies for HCC.

RNA-based therapeutics have emerged as a promising strategy for HCC treatment due to their ability to modulate gene expression directly associated with tumor progression, thus minimizing off-target effects and toxicity. RNA therapeutics, including microRNA (miRNA), small interfering RNA (siRNA), messenger RNA (mRNA), and small activating RNA (saRNA), offer a novel strategy for specifically modulating gene expression linked to tumorigenesis, facilitating precise and effective treatment 12-14. However, the clinical application of RNA therapeutics faces significant challenges due to the instability of RNA molecules, susceptibility to degradation by nucleases, poor cellular uptake, and potential immunogenicity.

Nanotechnology has provided solutions to many of these limitations by enabling the development of nanocarriers that protect and deliver RNA molecules effectively to tumor sites. Nanocarriers such as lipid nanoparticles (LNPs), polymeric nanoparticles (PNPs), and bioengineered vectors enhance the stability, bioavailability, and targeted delivery of RNA molecules 15-18. LNPs, for example, protect RNA from enzymatic degradation and facilitate efficient cellular uptake, as demonstrated in the delivery of mRNA vaccines during the COVID-19 pandemic 19. Similarly, PNPs and extracellular vesicles (EVs) can be engineered to improve delivery efficiency, reduce off-target effects, and modulate the tumor microenvironment (TME) to enhance therapeutic outcomes. For example, by functionalizing the nanoparticle surface with tumor-specific ligands or antibodies, the nanoparticles can selectively bind to receptors overexpressed on HCC cells, such as glypican-3 (GPC3) or transferrin receptors 20, 21. This targeted binding facilitates increased accumulation of the therapeutic RNA at the tumor site, improving efficacy while reducing systemic toxicity and side effects. Additionally, this strategy allows for controlled release of the RNA payload in response to specific stimuli within the TME, such as pH changes or enzymatic activity, further enhancing therapeutic precision.

Therefore, RNA nanotherapeutics, which involve nanocarrier-encapsulated RNA therapeutics, have shown significant progress in HCC treatment. The first approved siRNA drug, Patisiran, which is formulated into LNPs, effectively treats hereditary transthyretin-mediated amyloidosis, and its liver-specific delivery system also shows potential for liver cancer treatment 22. TKM-PLK consists of siRNA designed to silence PLK1 encapsulated in LNPs, and is undergoing clinical trials for HCC treatment 23. miRNA-based therapies have also entered clinical trials, with MRX34 being the first therapy to target liver cancer 24. saRNA has also demonstrated promising potential in liver cancer therapy; the first saRNA drug to enter clinical trials, MTL-CEBPA (NCT02716012), has shown excellent safety and therapeutic prospects when combined with TKIs, potentially becoming the first RNA drug for HCC treatment 25. Moreover, our group was the first to demonstrate the therapeutic potential of restoring tumor suppressor function using RNA-based therapeutics in liver cancer, initially demonstrating that p53 restoration sensitizes p53-deficient cancers to mTOR inhibition and subsequently showing that p53 restoration enhances anti-tumor immunity 26. More recently, we reported a PNP platform for delivering p53 mRNA, which directly inhibited liver cancer growth and enhanced anti-tumor immune responses by modulating the immune microenvironment. When this mRNA delivery system was combined with immune checkpoint inhibitors such as anti-PD-1, it significantly enhanced anti-tumor effects, surpassing the efficacy of anti-PD-1 therapy alone 27. These developments underscore the versatility of RNA nanotherapeutics, which hold considerable promise for overcoming the challenges of conventional HCC therapies through targeted, potent, and personalized approaches.

**Figure 1:**
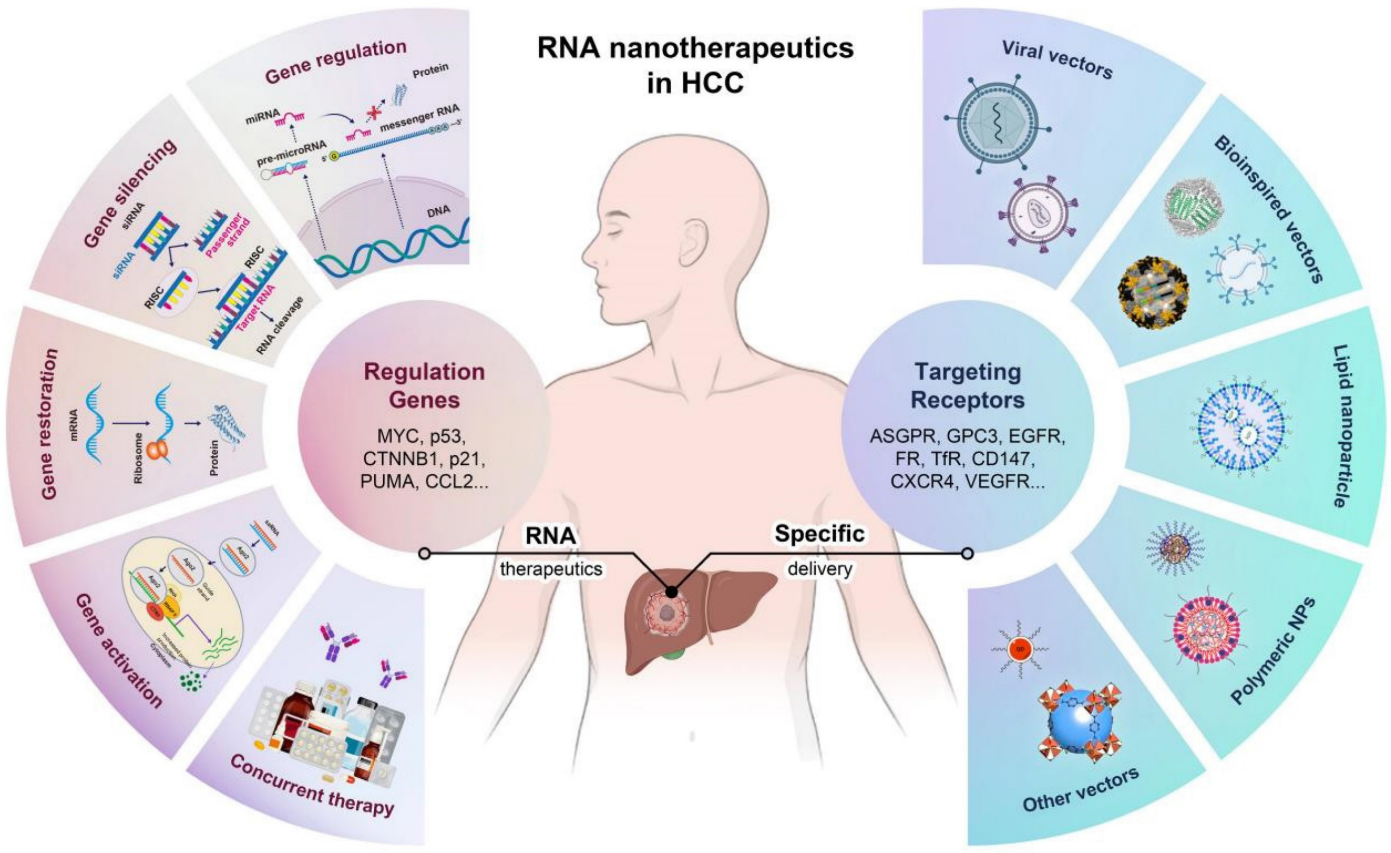
Schematic of RNA nanotherapeutics in HCC treatment. This schematic illustrates the multifaceted approach of RNA-based therapies in HCC, including gene silencing, regulation, restoration, activation, and concurrent therapy. The left side highlights various RNA strategies, including gene silencing, gene regulation, gene restoration, gene activation, and concurrent therapy, targeting critical genes such as MYC, p53, and CTNNB1. The right side shows the advanced nanocarrier systems used for a specific delivery, including viral vectors, bioinspired vectors, LNPs, PNPs, and other NPs, to target HCC receptors like ASGPR, GPC3, and EGFR, enhancing the precision of RNA delivery to HCC.

The rationale for using nanotechnology-enhanced RNA therapeutics in HCC allows for precise modulation of genes involved in hepatocarcinogenesis, offering potential for more effective and personalized treatment options 28-30. In this review, as shown in **Figure 1**, we summarize the concept of RNA nanotherapeutics in HCC, showcasing key approaches such as gene regulation, gene silencing, gene restoration, gene activation, and concurrent therapies. Different RNA types, including siRNA, miRNA, and mRNA, are being utilized to target genes involved in HCC progression. We also illustrate the various delivery vectors, such as bioinspired vectors, LNPs, PNPs, and other vectors, used to target receptors overexpressed on HCC cells (e.g., ASGPR, GPC3, EGFR, and CXCR4). The convergence of RNA-based strategies and nanotechnology not only paves the way for overcoming the multifaceted resistance observed in HCC treatment, but also aligns with the overarching goal of precision medicine in oncology. This review explores the current state and prospects of RNA-based therapies in treating HCC, highlighting the advances, challenges, and potential of this emerging field, and providing new directions for designing precise, effective, and personalized RNA nanomedicines.

## Types and mechanisms of RNA therapeutics

**Figure 2:**
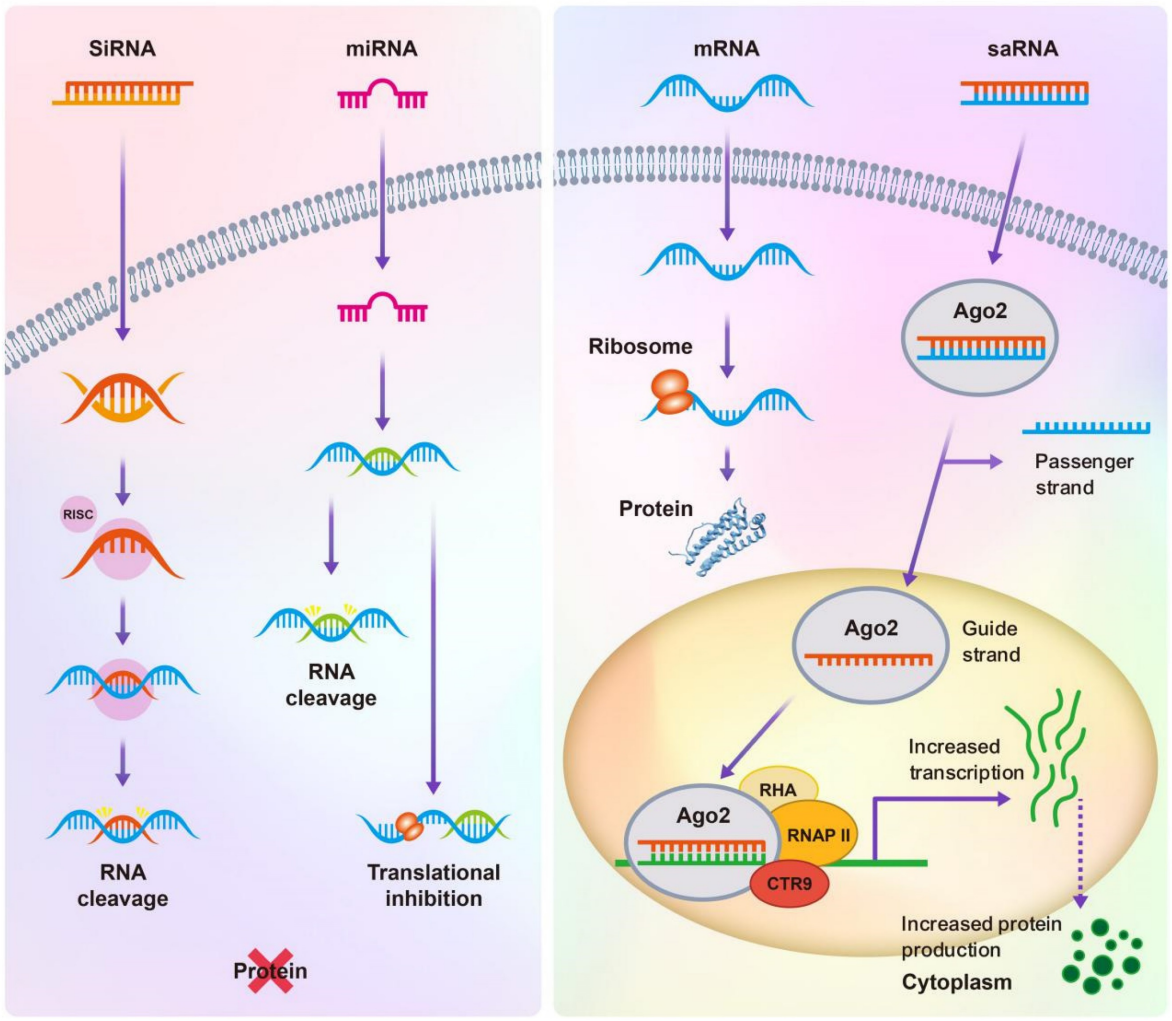
Types and mechanisms of RNA-based therapeutics. siRNA and miRNA induce gene silencing through mRNA cleavage or translational inhibition, while mRNA and saRNA enhance protein production by direct translation or transcriptional activation.

RNA therapies have emerged as a revolutionary force in modern medicine, distinguished by their precision, versatility, and safety, making them ideal for treating a wide array of diseases, from genetic disorders to cancer. These therapies precisely target specific genes or pathways, significantly reducing potential side effects by affecting only disease-related processes. Diverse RNA molecules, including miRNA, siRNA, mRNA, and saRNA, provide various mechanisms for gene silencing, expression enhancement, or protein replacement (**Figure 2**), allowing RNA therapies to adapt to various medical conditions, from genetic diseases to cancer. Moreover, the rapid development cycle of RNA molecules enables swift responses to emerging health challenges, such as viral outbreaks. Importantly, unlike some gene therapies that may integrate into the genome and potentially cause mutations, RNA therapies do not integrate into the host DNA, thereby avoiding the risks of long-term genetic alterations and enhancing their safety profile 31. Additionally, RNA therapies allow for the control of gene expression duration and intensity, enabling personalized treatment approaches that ensure efficacy while minimizing risks 32. Overall, these characteristics underscore the transformative potential of RNA therapies in advancing the capabilities of precision medicine.

### MicroRNA (miRNA)

miRNA is a class of small non-coding RNA molecules that play a critical role in gene expression regulation through the RNA interference pathway. Typically around 22 nucleotides in length, miRNAs are initially transcribed from DNA as longer primary transcripts (pri-miRNAs) and are then processed into shorter precursor miRNAs (pre-miRNAs) in the nucleus. The pre-miRNA is exported to the cytoplasm, which is further processed by the Dicer enzyme into mature miRNA duplexes. One strand of the duplex, the mature miRNA, is incorporated into the RNA-induced silencing complex (RISC), guiding the complex to complementary mRNA targets, thereby inhibiting their expression through mRNA degradation or translational repression 33. miRNAs are crucial for maintaining cellular homeostasis, and their dysregulation is associated with various diseases, including cancer, cardiovascular diseases, and neurodegenerative disorders 34. This makes them valuable targets for research as diagnostic and prognostic biomarkers and therapeutic agents. In oncology, specific miRNAs differentiate tumor types based on their expression profiles. Therapeutically, miRNAs can be manipulated to inhibit tumor growth by modulating the expression of oncogenes or tumor suppressor genes 35.

The use of miRNA for therapy offers several advantages, including high specificity and the ability to simultaneously regulate multiple targets involved in disease pathways, potentially leading to more comprehensive and effective treatment outcomes. Their small size and natural presence in the body reduce the likelihood of adverse immune reactions, which is a significant advantage over other therapeutic agents. However, miRNA-based therapies also face challenges. Delivering miRNAs to specific cells and tissues without degradation remains a major obstacle due to their instability in the bloodstream and the general inefficiency of existing delivery systems 36. Additionally, miRNAs may inadvertently regulate unintended mRNA targets, leading to off-target effects 37. Given their complex regulatory roles in various cellular processes, altering miRNA levels may have widespread and unpredictable impacts on other biological processes.

The miRNA research continues to expand rapidly, offering new insights into gene regulation and cellular function. The development of novel delivery systems, such as NP-based carriers, and the refinement of miRNA mimics and inhibitors, are advancing the clinical application of miRNAs. As our understanding of miRNAs deepens and technology progresses, miRNAs are expected to become a central component of targeted therapeutic strategies, offering solutions for diseases that currently lack effective treatments.

### Small interfering RNA (siRNA)

siRNA plays a crucial role in molecular biology and therapeutics, primarily through its function in the RNA interference (RNAi) pathway, which controls gene expression by silencing specific genes. Typically 20-25 nucleotides long, siRNAs are double-stranded molecules with two-nucleotide overhangs, formed from longer dsRNA precursors by the Dicer enzyme 38. The guide strand of siRNA is incorporated into the RISC, which binds to complementary mRNA, leading to its cleavage and degradation, thus silencing the gene.

In research, siRNAs are invaluable tools for studying gene function by selectively silencing genes. Therapeutically, siRNAs are being developed to treat conditions such as viral infections, cancer, and genetic disorders by targeting disease-related genes 39, 40. Their high specificity allows them to minimize off-target effects compared to traditional drugs. However, challenges remain in delivering siRNAs effectively into cells while maintaining their stability in the bloodstream 41. There is also a risk of siRNAs triggering immune responses, as they can resemble viral RNA.

Despite these challenges, significant progress has been made in the clinical development of siRNA-based gene therapies. Techniques to improve siRNA stability and delivery, such as chemical modifications and NP carriers, have advanced to clinical trials. For example, Patisiran, a siRNA therapeutic targeting transthyretin, has been approved by the FDA for treating hereditary transthyretin-mediated amyloidosis, marking a significant milestone in RNAi therapy 42. In summary, siRNA technology offers a powerful means of gene therapy, with the potential to precisely alter gene expression and address diseases at their genetic roots.

### Messenger RNA (mRNA)

mRNA therapy is a revolutionary approach in medicine, leveraging the body's cellular machinery to produce therapeutic proteins. This strategy has significantly advanced research and clinical applications, particularly in addressing diseases rooted in genetic abnormalities or protein dysfunction 43-45. Although the concept of using mRNA as a therapeutic tool emerged decades ago, it was only in the late 20th and early 21st centuries that technological breakthroughs enabled the development of stable and effective mRNA therapies 46. Early challenges, such as mRNA instability and high immunogenicity, initially hindered its therapeutic potential 47. However, advancements in modified nucleotides have reduced these issues, paving the way for mRNA's use in vaccines and protein replacement therapies. mRNA, a single-stranded molecule, carries genetic instructions from DNA to ribosomes, where it directs protein synthesis. Therapeutically, synthetic mRNA is designed to encode specific proteins, and once delivered, it utilizes the cell's machinery to produce these proteins. This technology's flexibility has led to its exploration in vaccines, cancer immunotherapy, and genetic disorder treatments. The success of mRNA vaccines in combating COVID-19, particularly through the Pfizer-BioNTech and Moderna vaccines, highlights its potential 48. Beyond infectious diseases, mRNA is being investigated for its ability to stimulate immune responses against cancer by encoding tumor antigens, and in genetic disorders 49-51, it serves as a template for producing missing or defective proteins.

A key advantage of mRNA therapy is its safety, as mRNA does not integrate into the host genome, eliminating the risk of insertional mutagenesis associated with some gene therapies. mRNA therapy is also highly flexible, allowing for rapid design and manufacturing, which is particularly beneficial in responding to emerging health threats and pandemics. However, mRNA therapy also faces challenges, primarily related to delivery and stability 52. mRNA molecules are inherently unstable and prone to degradation, requiring sophisticated delivery systems to protect the mRNA and ensure its effective uptake and expression in target cells. Common strategies include encapsulating mRNA in LNPs to enhance cellular uptake and protect it from degradation. While mRNA's immunogenicity is advantageous in vaccine applications, it can be a drawback in other therapeutic contexts, potentially triggering unwanted immune responses 53.

The success of mRNA COVID-19 vaccines has catalyzed further research into broader applications. Continued research is focused on refining mRNA modifications and delivery systems to enhance the safety, efficacy, and durability of mRNA therapies 54. In summary, mRNA therapy presents a dynamic and adaptable platform in modern medicine, offering significant potential across a broad spectrum of diseases. As understanding and technologies improve, mRNA is set to become a cornerstone in the future of therapeutic strategies.

### Small activating RNA (saRNA)

saRNA represents an innovative class of RNA therapies distinct from the more common silencing RNA. Unlike siRNA, which is designed to downregulate gene expression, saRNA is engineered to upregulate gene expression 55, 56. This unique capability opens new avenues for therapeutic intervention, particularly in cases where gene activation is required. The concept of saRNA emerged from the RNAi field, which traditionally focused on gene silencing. The discovery that RNA could also be used to enhance gene expression significantly altered the initial understanding of RNAi 57. Early studies showed that double-stranded RNA could target promoters and enhance gene transcription, a phenomenon first observed in plants and later confirmed in mammalian cells. This discovery led to the identification and development of saRNA as a new tool for gene activation.

saRNAs are short, double-stranded RNA molecules (20-25 nucleotides) that target promoter regions to recruit RNAi machinery and transcription factors, enhancing gene transcription 58. This unique ability makes saRNA valuable for diseases requiring gene activation, such as liver diseases and metabolic disorders, by promoting the production of proteins that reverse liver damage or regulate metabolic pathways 59. saRNA also holds potential in regenerative medicine, such as activating genes involved in cell growth and differentiation 60, 61.

Compared to other RNAi technologies, the clinical development of saRNA is still in its early stages. However, promising preclinical studies have laid the foundation for initial clinical trials. For example, an saRNA designed to activate the CEBPA gene, a crucial regulator in bone marrow biology, is being investigated for treating liver cancer and certain hematologic disorders 62, 63. This reflects the broad potential of saRNA in treating a wide range of diseases that require gene activation for effective therapy.

saRNA's ability to selectively activate genes broadens the therapeutic landscape beyond traditional silencing approaches. It can be synthesized using existing RNAi technology platforms, facilitating rapid development. However, challenges remain: ensuring consistent and efficient gene activation requires precise targeting 64. Like other RNA therapies, saRNA also faces delivery challenges, requiring protection from degradation and effective cellular uptake mechanisms to reach its target sites in the genome 65. Therefore, saRNA represents a promising yet still evolving technology in RNA therapies. With its unique ability to activate gene expression, saRNA has the potential to play a significant role in treating a wide range of genetic and acquired diseases. Continued research and development are essential to overcoming current limitations and fully realizing the clinical potential of this innovative therapy.

## Nanotechnology improves RNA delivery

Effective RNA therapy depends on the precise and stable delivery of RNA to target cells and its successful release from lysosomes or endosomes. NPs play a critical role in protecting RNA molecules from degradation in the bloodstream, enhancing their stability and lifespan. These NPs can be functionalized with ligands or antibodies on their surface to recognize specific cellular receptors, creating delivery systems tailored to target specific tissues or cell types. This targeted delivery improves RNA uptake by diseased cells while minimizing off-target effects. Moreover, NPs can be engineered to respond to specific stimuli, ensuring controlled release in the target environment. This approach maximizes therapeutic efficacy while reducing side effects. Additionally, NPs offer multifunctionality, integrating therapeutic and diagnostic capabilities within a single platform, enabling real-time monitoring of treatment and disease progression 66. The combination of RNA therapies with advanced NP delivery systems marks a significant advancement in precision medicine, offering highly effective, customizable, and safe treatment options.

**Figure 3:**
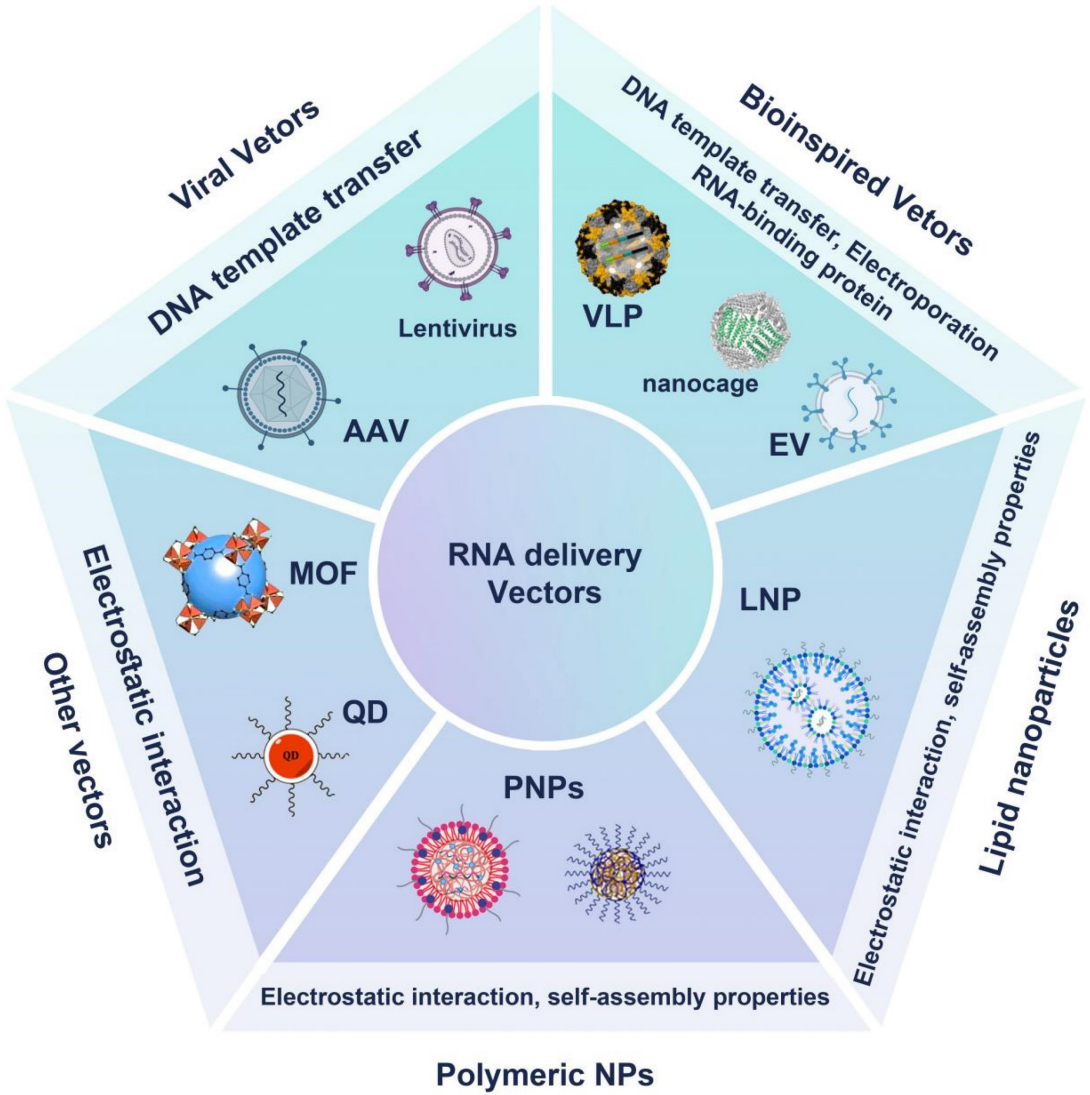
Overview of RNA delivery vectors and their underlying mechanisms. Viral vectors, such as lentivirus and AAV, rely on DNA template transfer to facilitate RNA delivery; Bioinspired vectors, including VLP, EV, and nanocages, utilize mechanisms like DNA template transfer, electroporation, and RNA-binding proteins; LNPs and PNPs achieve RNA delivery through electrostatic interactions and self-assembly properties to enhance cellular uptake; Other vectors, including MOF and QD, rely on electrostatic interactions to facilitate RNA binding and delivery.

The efficient delivery of RNA molecules relies on advanced nanocarrier systems, each employing unique mechanisms to ensure stability and specificity. As shown in the **Figure 3**, various RNA delivery vectors have been developed to optimize therapeutic efficacy. Gene delivery vectors include viral options like lentiviruses, and adeno-associated viruses (AAV), which are efficient but may trigger immune responses and pose risks of insertional mutagenesis 67; Non-viral alternatives include bioinspired NPs such as virus-like particles (VLPs), nanocages, EVs, and exosomes 68, which mimic biological structures to enhance biocompatibility. LNPs are particularly noteworthy in RNA therapy, providing protection and improving cellular uptake efficiency, while PNPs made from materials like PLGA allow for controlled, stimulus-responsive release 69, 70. Other innovative materials like metal-organic frameworks (MOFs) and quantum dots (QDs) offer unique properties for RNA delivery and therapeutic tracking 71.

### Viral vectors

Viral vectors are powerful tools in gene therapy, widely used for RNA delivery due to their inherent ability to enter cells and achieve high levels of gene expression. These vectors exploit the natural mechanisms viruses have evolved to introduce their genetic material into host cells. Viral vectors are engineered to be replication-deficient, meaning they cannot reproduce within the host cell, thereby significantly reducing their pathogenicity 72-74. Commonly used viral vectors for RNA delivery include lentiviruses, adenoviruses, AAV, and retroviruses, each with unique characteristics suitable for specific applications. For example, lentiviruses integrate into the host genome, allowing long-term expression in both dividing and non-dividing cells, while adenoviruses achieve high transduction efficiency but provide only transient expression as they do not integrate into the genome.

Viral vectors have driven the development of numerous clinical applications. For example, AAV vectors are used in FDA-approved therapies like Luxturna™ for inherited retinal disease and Zolgensma™ for spinal muscular atrophy 75, 76, demonstrating their potential in treating genetic disorders. Lentiviral vectors have been successful in CAR-T cell therapies for cancer, where they modify T cells to target specific cancer cells 77. Research continues to explore the use of viral vectors in a wide range of conditions, including infectious diseases and metabolic disorders.

Despite their efficacy in delivering genetic material and achieving substantial gene expression, viral vectors come with significant challenges 78. Immunogenicity is a major issue, as the body's immune response to viral proteins can cause inflammation and reduce the therapy's effectiveness, particularly with repeated administration. Safety concerns also arise from the risk of insertional mutagenesis, especially with vectors like lentiviruses and retroviruses that integrate into the host genome. If these vectors insert themselves in a way that disrupts tumor suppressor genes or activates oncogenes, there is a potential cancer risk. Moreover, producing viral vectors is a complex and expensive process, demanding rigorous manufacturing standards to ensure safety and effectiveness.

### Bioinspired vectors

Bioinspired vectors for RNA delivery are an innovative class of delivery systems that mimic biological entities such as viruses and cellular components to facilitate the efficient and targeted delivery of RNA molecules. These vectors leverage biological systems' natural design and functional characteristics, providing a promising approach to overcoming challenges associated with synthetic delivery systems. Bioinspired vectors encompass a variety of structures, including VLPs, nanocages, EVs, and exosomes 18, 79, 80. VLPs are shell-like structures formed by engineered viral proteins that can encapsulate RNA, mimicking viral infection characteristics without the ability to replicate or cause disease. Nanocages are self-assembling protein sub-units ideal for encapsulating and protecting RNA molecules. EVs and exosomes are small vesicles naturally derived from cells that transport RNA and proteins through natural cellular pathways, facilitating targeted delivery and cellular uptake.

The progress in bioinspired vectors has been noteworthy in research and clinical contexts. VLPs have seen success in vaccine development, such as the hepatitis B vaccine, where they induce potent immune responses 81. Exosomes are being explored for cancer therapy, utilizing their ability to specifically target tissues and evade the immune system 82. Clinical trials are actively investigating the use of exosomes loaded with siRNA or miRNA to selectively alter gene expression in cancer cells 83, highlighting their potential in targeted cancer therapies.

The major benefits of bioinspired vectors include their high biocompatibility and low immunogenicity. Their natural or bioengineered origins generally make them more tolerable within the body, reducing the risk of adverse immune responses. Moreover, these vectors can leverage natural biological pathways for precise tissue targeting and efficient cellular uptake, crucial for minimizing off-target effects and maximizing therapeutic efficacy. Nevertheless, bioinspired vectors come with challenges, particularly in their production and purification, which can be complex and costly 84. The variability in the composition and size of naturally derived vectors like EVs and exosomes also presents difficulties in achieving consistent results, complicating standardization and regulatory approval. Additionally, despite their natural origin, patient-specific immune responses can vary, introducing further challenges in their application.

### Lipid nanoparticle vectors

LNPs are at the forefront of non-viral gene delivery systems, particularly noted for their success in the development of mRNA vaccines. Their unique structural and functional attributes allow for the efficient encapsulation and delivery of various nucleic acids. LNPs are typically composed of ionizable lipids that aid in encapsulating negatively charged nucleic acids, along with phospholipids, cholesterol, and polyethylene glycol (PEG)-modified lipids, which stabilize the NPs and extend their circulation time in the bloodstream 68, 69. The ionizable lipids are crucial as they enable LNPs to respond to the acidic environment of endosomes following cellular uptake, facilitating the release of RNA into the cytoplasm.

The most notable success of LNP technology has been in mRNA vaccines, particularly for COVID-19. Beyond vaccines, LNPs are also being explored to deliver gene-editing tools like CRISPR-Cas9 and treat genetic diseases by delivering therapeutic mRNA or siRNA. For example, Onpattro, an LNP-formulated siRNA therapy, has been approved for treating hereditary transthyretin-mediated amyloidosis 85, showcasing the clinical viability of LNP-based therapies.

The most notable concerns of LNPs are the potential immunogenicity and inflammatory responses associated with the ionizable lipids and PEG components. Managing these responses is crucial for the safety and efficacy of LNP-based therapies. Additionally, producing LNPs requires sophisticated technology to ensure consistent quality and properties, which can be costly and technically demanding.

### Polymer nanoparticle vectors

PNPs are a versatile class of carriers used for RNA delivery, made from biodegradable polymers that can encapsulate and protect RNA molecules while facilitating their entry into target cells. These NPs are gaining increasing attention for their potential in precision medicine, particularly in gene therapy and vaccine development. PNPs are typically made from synthetic polymers such as poly(lactic-co-glycolic acid) (PLGA), polyethylenimine (PEI), or natural polymers like chitosan 86, 87. These materials are chosen for their biocompatibility and biodegradability, allowing the polymers to safely degrade into non-toxic byproducts that can be easily cleared from the body. Polymers can be engineered into various shapes and sizes and can be functionalized with targeting ligands or modified to respond to specific environmental triggers 88, such as pH changes, enhancing the release of RNA payloads into the target cytoplasm.

The study of PNPs for RNA delivery has significantly expanded, exploring their applications in gene silencing, mRNA-based vaccines, and CRISPR-Cas9 gene editing systems. In clinical settings, PNPs are being investigated in numerous trials, particularly in cancer treatment, where siRNA is used to silence oncogenes. For example, Calando Pharmaceuticals developed a PNP (CALAA-01) that delivers siRNA targeting the M2 subunit of ribonucleotide reductase, showing promising results in solid tumors 89. In vaccines, PNPs are being explored for their ability to efficiently deliver mRNA vaccines, aiming to enhance immune responses while minimizing adverse effects.

The main advantages of PNPs include their protective and controlled release capabilities, which ensure that RNA therapies are effectively delivered and remain stable until they reach the target site 124. Polymer modifiability allows for precise tuning of NP properties, such as degradation rate, mechanical strength, and interactions with biological barriers, making them highly adaptable to various therapeutic needs. Additionally, by attaching specific ligands to their surface, PNPs can be designed to target specific tissues or cell types, enhancing the specificity and potency of RNA delivery.

However, challenges associated with PNP vectors also exist 90. A primary concern is the potential cytotoxicity, particularly when using cationic polymers like PEI, which may disrupt cell membranes and lead to inflammatory responses. Additionally, the variability in the synthesis of PNPs can lead to batch-to-batch inconsistencies, posing scalability and clinical application challenges. Designing polymers that degrade at an appropriate rate to effectively release their payloads without premature degradation or prolonged retention in the body is another significant technical hurdle.

### Other vectors

In addition to the more commonly used lipid and PNPs, other NP vectors for RNA delivery include a diverse range of materials such as MOFs, QDs, and silica-based NPs. These carriers offer unique properties that can enhance the delivery, stability, and targeting of RNA therapies, making them valuable tools in advancing gene therapy.

As exemplified by ZIF-8, MOFs are crystalline frameworks composed of metal ions and organic ligands, offering large surface areas and customizable porosity for effective RNA encapsulation 91. QDs, primarily recognized for their fluorescent properties, are now being adapted for RNA delivery 92, providing the dual benefit of therapeutic delivery and real-time imaging. Silica-based NPs, especially mesoporous variants, are valued for their large surface areas and chemical stability, which are ideal for RNA loading and controlled release 93. Preclinical studies have demonstrated the potential of these vectors. For instance, ZIF-8 MOFs have shown promise in RNA protection and gene silencing in cancer models. QDs are being explored to track RNA therapies while delivering therapeutic payloads. Silica-based NPs have effectively delivered siRNA for lung cancer, improving RNA stability and targeted release. While MOFs, QDs, and silica NPs are still in the experimental phase, their unique capabilities present exciting possibilities for RNA delivery in gene therapy. Continued research and development will be crucial in addressing their limitations and unlocking their full potential in clinical applications.

Various vector types are employed to enhance the delivery, stability, and specificity of gene therapies. Each vector type offers distinct advantages and faces unique challenges, making the choice of vector crucial for the success of therapeutic applications. **Table 1** provides a comparative overview of different vectors used in gene delivery, highlighting their key features, benefits, and limitations. This comparison aims to guide the selection of appropriate delivery systems for RNA-based therapies in HCC and other diseases.

## Characteristics of the liver and HCC

Before delving into the innovative realm of RNA nanomedicine for liver cancer therapy, it is crucial to comprehensively elucidate the structural and functional characteristics of the liver and HCC. This section examines the liver's unique anatomical structure and functional attributes, detailing its pivotal roles in metabolism, detoxification, and immune regulation, all essential for optimizing RNA nanotherapeutic strategies. Additionally, it explores the specific pathophysiological features of liver cancer, particularly HCC, including the TME and molecular heterogeneity that influence the efficacy of therapeutic interventions. Understanding these characteristics is fundamental to the development of RNA nanomedicines that can effectively target and treat liver cancer, providing a theoretical foundation for enhancing therapeutic efficiency and precision.

### Liver structure and function

As the largest visceral organ, the liver plays a crucial role in numerous physiological processes vital for survival and health. Beyond its metabolic functions, the liver supports immune responses by eliminating pathogens and exogenous antigens, underscoring its status as the body's largest metabolic organ 94. Its complex structural and functional framework is key to the development of targeted RNA nanotherapeutics. The liver's extensive vascular network, with dual blood supply from the hepatic artery and portal vein, not only facilitates nutrient and drug delivery but also significantly influences the distribution and metabolism of therapeutic agents, including RNA nanotherapeutics 95. The liver's anatomical complexity, with lobules containing hepatocytes and non-parenchymal cells such as Kupffer cells, hepatic stellate cells, and liver sinusoidal endothelial cells (LSECs), is central to understanding targeted RNA delivery. Hepatocytes are involved in metabolism and protein synthesis, while LSECs and Kupffer cells modulate immune and endocytic functions, offering specific targets for RNA therapies 96-99. These extensive functional capacities are highly relevant to RNA therapies, which often aim to modulate specific metabolic pathways associated with diseases like HCC.

Given the liver's filtering capacity and complex microenvironment, designing RNA nanomedicines requires specific adaptations to enhance delivery efficiency. For instance, leveraging the high endocytic activity of LSECs can enhance the efficiency of RNA delivery systems, and specific cellular interactions within the liver microenvironment can be exploited to improve the uptake and efficacy of therapeutic RNA 100. Recent studies have shown that after entering the liver, PEGylated liposomal drugs are first taken up by Kupffer cells, where the liposomes are degraded intracellularly, releasing free drugs that evade LSEC capture and directly enter hepatocytes 101. The amount of liposomal drug captured by Kupffer cells positively correlates with subsequent drug accumulation in hepatocytes. These highlight the importance of understanding liver-specific uptake mechanisms to optimize nanoparticle formulations for HCC. Advanced strategies for targeted delivery in the liver aim to improve stability, enhance tumor specificity, and avoid rapid metabolism or clearance, thus maximizing therapeutic efficacy and minimizing side effects 102, 103.

### Structural and microenvironmental features of HCC

The TME of HCC is highly complex and plays a central role in disease progression and therapy response. HCC often exhibits diverse morphological features, ranging from well-differentiated to poorly differentiated, due to intratumoral heterogeneity 104. This variability contributes to its aggressive behavior and challenges therapeutic efficacy, as tumor regions differ in vascularization and cellular composition 105, 106. Unlike the finely organized vasculature of normal liver tissue, HCC often exhibits disorganized and unstructured vascularization. This abnormal vasculature leads to hypoxic conditions within the tumor, driving further genetic instability and tumor progression 107, 108. Arteriovenous shunting and sinusoidal endothelial cell capillarization are typical findings in HCC 109, complicating drug delivery.

Moreover, the TME of HCC comprises various cell types, including immune cells (T cells, macrophages), fibroblasts, and endothelial cells, along with the extracellular matrix (ECM) 110. These components interact dynamically, creating either pro-tumor or anti-tumor conditions. Chronic inflammation in HCC, caused by precursor conditions like hepatitis or cirrhosis, fosters an immunosuppressive TME, making effective treatment challenging 111. For example, Regulatory T cells (Tregs), myeloid-derived suppressor cells (MDSCs), and cancer-associated fibroblasts (CAFs) all play roles in promoting immune evasion and tumor progression 112-114.

Understanding the dynamics of the HCC TME is crucial for designing effective RNA-based therapies. By targeting specific components of the TME, RNA nanotherapeuitcs can modulate immune suppression, promote immune activation, and inhibit critical pathways involved in tumor progression 115. RNA nanotherapeutics can be designed to silence genes critical for tumor cell survival and proliferation or to modulate the immune environment, enhancing the presentation of tumor antigens and restoring the activity of effector immune cells to improve therapeutic outcomes 116. This comprehensive understanding of liver and TME features allows for the development of targeted strategies that leverage tumor vulnerabilities for more effective treatment outcomes.

### Strategies for targeted delivery in HCC

The effective delivery of RNA nanotherapeutics for HCC necessitates a thorough understanding of the liver's unique anatomy, physiological characteristics, and the complex TME specific to HCC. To enhance specificity while minimizing off-target effects and systemic toxicity, advanced delivery strategies have been developed. The following subsections provide an in-depth exploration of these approaches, each designed to address the unique challenges and take advantage of the liver's and HCC's microenvironmental characteristics.

#### Utilizing HCC-specific receptors for improved tumor selectivity

Targeting hepatocellular carcinoma-specific receptors is a critical strategy for enhancing the selectivity of RNA nanomedicine delivery, thereby improving treatment efficacy while minimizing side effects. GPC3 is a highly promising target for HCC-specific delivery, as it is overexpressed on HCC cells but largely absent in normal liver tissue 117. Antibody-functionalized nanoparticles, such as those modified with anti-GPC3 antibodies, have demonstrated significant efficacy in delivering RNA molecules directly to HCC cells, allowing for targeted silencing of oncogenic pathways or activation of tumor suppressor genes. Preclinical studies have shown that such nanoparticles can selectively accumulate in GPC3-positive tumors, reducing the risk of off-target effects in healthy liver tissues.

Asialoglycoprotein receptor (ASGPR) is another critical target for enhancing selectivity. ASGPR is predominantly expressed on hepatocytes, and leveraging ligands such as N-acetylgalactosamine (GalNAc) enables efficient receptor-mediated endocytosis of nanoparticles carrying RNA payloads. This receptor is already being utilized successfully in clinical settings, such as in Inclisiran, an siRNA therapeutic targeting cholesterol synthesis via hepatic ASGPR uptake. This approach demonstrates the potential for GalNAc-functionalized nanoparticles in delivering RNA therapeutics specifically to the liver, effectively bypassing non-hepatic tissues 118. Similarly, the transferrin receptor, which is overexpressed in many cancers including HCC, has been utilized to improve nanoparticle uptake by tumor cells. Transferrin-functionalized nanoparticles carrying siRNA or miRNA have shown increased selectivity and uptake by HCC cells, as HCC tends to have a higher demand for iron compared to healthy cells 119. This not only enhances the efficacy of the therapeutic RNA but also reduces the likelihood of systemic side effects, making transferrin a valuable ligand in targeted nanoparticle design 120. Folate receptors (FR) and epidermal growth factor receptor (EGFR) are also promising targets for HCC-specific delivery 121. While folate receptors are expressed in more aggressive HCC subtypes, EGFR is frequently upregulated in HCC, particularly in poorly differentiated tumors. Anti-EGFR-modified nanoparticles and folate-functionalized carriers have been effectively utilized to deliver siRNA or mRNA to suppress tumor growth, demonstrating improved outcomes in HCC models.

#### Leveraging liver fenestrations for enhanced nanoparticle delivery

The fenestrated architecture of LSECs offers a unique avenue for enhancing nanoparticle delivery 122. LSECs contain fenestrations, transcellular pores approximately 50-150 nm in size, that facilitate the movement of substances, including nanoparticles, from the bloodstream into the liver parenchyma. This fenestrated structure allows for the passive accumulation of nanoparticles in the liver, provided they are appropriately designed in terms of size and surface characteristics. Optimizing the size of nanoparticles to match the dimensions of LSEC fenestrations allows for effective translocation to hepatocytes or HCC cells. LNPs and PNPs, when sized appropriately (generally below 100 nm), can readily traverse LSEC fenestrations and accumulate in hepatic tissues 123. Recent advancements in size-tunable polymeric micelles have demonstrated improved delivery to the liver by adjusting nanoparticle size to optimally pass through the fenestration 124. Such size optimization is critical to ensuring enhanced hepatic accumulation while minimizing non-specific distribution in other organs.

#### Overcoming the TME barriers for efficient delivery

The TME of HCC poses significant challenges for effective therapeutic delivery, characterized by a dense ECM, abnormal vasculature, and hypoxic regions 125. To overcome these barriers, advanced strategies are being employed to enhance the efficacy of RNA nanomedicines. The enhanced permeability and retention (EPR) effect, resulting from leaky vasculature in tumors, facilitates the passive accumulation of nanoparticles in the tumor tissue, which provides a foundational mechanism for RNA-based nanomedicines.

Beyond passive targeting, pH-sensitive and hypoxia-responsive nanoparticles have been developed to enhance delivery under specific TME conditions 126. These nanoparticles are stable during circulation but are designed to release their RNA payload under the acidic or hypoxic conditions found within HCC tumors. To address the challenge of dense ECM, nanoparticles can be functionalized with hyaluronidase, which degrades the ECM components, thereby enhancing nanoparticle penetration and uniform distribution throughout the tumor. This enzyme-functionalization approach enables nanoparticles to reach poorly vascularized regions within the tumor, ensuring more comprehensive coverage and efficacy.

Furthermore, Kupffer cells and LSECs, which play key roles in nanoparticle uptake and biodistribution. Kupffer cells, the resident macrophages of the liver, are responsible for filtering out foreign particles from the blood. PEGylated liposomes, for example, are taken up by Kupffer cells, where they are subsequently degraded to release the active RNA payloads, which may then reach hepatocytes and tumor cells 101. This sequential process allows for efficient accumulation of therapeutic agents within hepatic tissues. In addition, LSECs possess high endocytic activity, which can be leveraged to facilitate RNA nanoparticle uptake 127. Functionalizing nanoparticles with positively charged peptides or cationic lipids enhances their interaction with LSECs, leading to improved internalization and delivery to HCC cells or hepatocytes. By optimizing these cell-specific interactions, nanoparticles can achieve more efficient hepatic and tumor-specific delivery.

#### Enhancing biocompatibility and minimizing off-target effects

Biocompatibility and minimizing immune responses are crucial for the success of RNA-based therapies in HCC. Biomimetic carriers, such as EV-like particles or cell membrane-coated nanoparticles, are increasingly being utilized to enhance biocompatibility. These biomimetic nanoparticles exhibit reduced immunogenicity due to their natural origin, allowing them to evade immune recognition and prolong systemic circulation, which is essential for successful repeated administration. Also, the biomimetic carriers could be further engineered with HCC targeting ligand to enhance the HCC targeting efficacy, such as our team reported that transferrin displayed 293T cell membrane-coated EV and NGR peptide expressed red blood cell membrane-coated EV exhibited much higher HCC accumulation than the non-engineered EV 128, 129.

In addition to biomimetic strategies, stimuli-responsive nanoparticles have been developed that release their RNA payload only upon exposure to specific conditions found in the TME, such as an acidic pH or the presence of enzymes like matrix metalloproteinases (MMPs) 130. This specificity allows for the precise targeting of HCC cells while minimizing damage to normal liver cells. MMP-responsive nanoparticles carrying siRNA or mRNA targeting tumor growth factors have shown efficacy in preclinical HCC models by releasing their cargo in the presence of elevated MMPs, a characteristic feature of the HCC TME.

The unique anatomical and physiological features of the liver, coupled with the complex TME in HCC, present challenges and opportunities for targeted delivery of RNA-based nanomedicines. By focusing on HCC-specific receptors for tumor selectivity, utilizing liver fenestrations for effective nanoparticle translocation, overcoming TME barriers through advanced nanoparticle designs, leveraging specialized liver cell functions, and enhancing biocompatibility, RNA nanotherapeutics hold significant promise for precise and effective HCC treatment. These strategies represent major advancements over traditional therapeutic approaches, providing targeted, safe, and efficient treatment options for HCC patients.

## Applications of RNA nanotherapeutics for HCC treatment

RNA nanotherapeutics offer a promising approach for treating HCC, leveraging advanced NP delivery systems to target and regulate key genetic pathways associated with cancer progression. These therapies include siRNA for gene silencing, miRNA modulation, and mRNA and saRNA for protein expression, all delivered through NPs designed to enhance stability, cellular uptake, and targeted delivery to HCC cells. By utilizing these technologies, RNA nanotherapeutics aim to overcome the limitations of traditional treatments, offering more precise, effective, and personalized treatment options for HCC patients.

### Gene regulation via miRNA nanotherapeutics

miRNA nanotherapeutics represents a sophisticated and emerging approach for treating HCC. miRNA nanotherapeutics based therapies aim to restore normal gene expression patterns in HCC, thereby inhibiting tumor growth and progression. In HCC, specific miRNAs can function as either tumor suppressors or oncogenes. Over the past decade, significant progress in miRNA nanotherapeutics for HCC has been made, thanks to deeper insights into miRNA biology and advancements in delivery technologies.

One key example is miR-141, a tumor suppressor whose expression is often downregulated in HCC, leading to uncontrolled growth and metastasis 131, 132. Restoring miR-141 levels using a tumor-adhesive NPX-glue nanoparticle system effectively inhibits tumor growth by ensuring localized delivery at the tumor site, maximizing its therapeutic potential while minimizing side effects 133. Similarly, miR-122, a liver-specific miRNA often downregulated in HCC, is one of the most prevalent gene in HCC. The loss of miR-122 is linked to enhanced tumor growth, metastasis, and poor prognosis 134. Researchers have developed NP-based delivery systems to restore miR-122 levels in HCC models. For instance, LNPs encapsulating miR-122 mimics have effectively restored miR-122 levels in liver tumors, suppressing the expression of oncogenes like Cyclin G1 and ultimately reducing tumor growth and metastasis 135.

**Figure 4:**
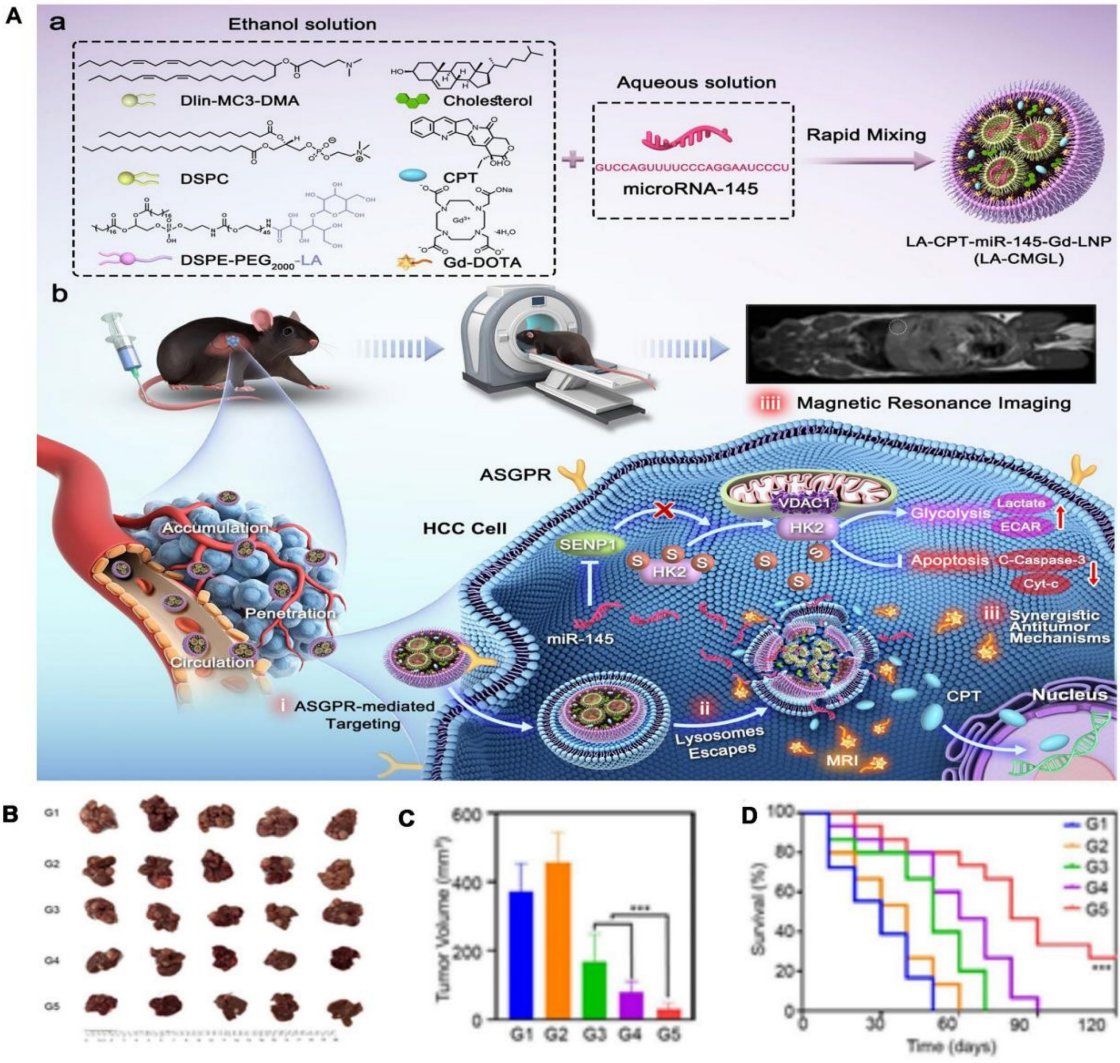
miRNA nanotherapeutics achieved HCC treatment. (**A**) Schematic and experimental validation of the LA-CPT-miR-145-Gd-LNP (LA-CMGL) system for targeted, MRI-visible therapy in HCC, and mechanism of ASGPR-mediated targeting, cellular uptake, lysosomal escape, and synergistic antitumor effects in HCC cells, alongside MRI imaging capability for tracking accumulation. (**B**) Representative images of tumor samples across treatment groups. (**C**) Tumor volume comparison indicating enhanced efficacy of LA-CMGL. (**D**) Survival analysis showing improved outcomes in treated groups compared to controls. Adapted from 136. Copyright 2024, Springer Nature.

Another crucial miRNA is miR-145, which acts as a tumor suppressor by inhibiting pathways involved in cell proliferation and metastasis. As shown in **Figure 4**, the study utilizes a lipid nanoparticle system, LA-CPT-miR-145-Gd-LNP (LA-CMGL), co-loaded with camptothecin (CPT) and miR-145, designed for targeted delivery to HCC cells 136. The nanoparticles are modified with lactobionic acid (LA) to target ASGPR overexpressed on HCC cells, enhancing cellular uptake through ASGPR-mediated endocytosis. Upon delivery, miR-145 exerts its tumor-suppressive effects by inhibiting oncogenic pathways, while CPT induces apoptosis, resulting in a synergistic antitumor effect. Additionally, Gd-DOTA provides MRI visibility, allowing for real-time monitoring of nanoparticle accumulation in tumors. This theranostic approach demonstrates significant tumor growth suppression and extended survival in HCC models, highlighting the potential of miR-145 in combination with chemotherapy for effective HCC therapy. These preclinical successes have spurred interest in clinical trials for miR-122-based therapies.

The clinical translation of miRNA nanotherapeutics is gaining momentum, with several candidates entering early-stage clinical trials. A notable example is MRX34, a liposomal formulation of miR-34a mimic, targeting multiple oncogenes involved in HCC progression, including MYC and BCL-2. MRX34 was the first miRNA mimic to enter clinical trials for cancer treatment 137. Although early trials showed anti-tumor activity in patients with advanced HCC and other solid tumors, the trial was halted due to immune-related adverse events, highlighting the challenges of miRNA therapy and the need for further optimization of delivery systems and dosing regimens. Another promising candidate is miR-26a, which is downregulated in HCC. Restoring miR-26a through nanoparticle-based delivery, such as exosomes, has shown efficacy in reducing tumor burden by targeting Cyclin D2 and E2 138, 139.

Recent advancements in nanoparticle (NP) technology are further improving miRNA delivery. pH-sensitive NPs that release their payload in the acidic tumor environment, NPs that selectively bind to receptors overexpressed on HCC cells, and hybrid NPs combining multiple delivery strategies are all being developed to enhance specificity, stability, and therapeutic efficacy 140.

miRNA nanotherapeutics offer significant advantages over traditional therapies by regulating multiple genes simultaneously, thus addressing the complexity of HCC biology 141. NP delivery systems enhance the stability and bioavailability of miRNAs, ensuring their effective delivery to tumor sites while minimizing off-target effects. Additionally, miRNA therapy can be customized based on the individual patient's characteristics, providing a more effective and less toxic personalized treatment strategy.

Overall, miRNA nanotherapeutics illustrate a promising and innovative approach for treating HCC by leveraging the regulatory potential of miRNAs to modulate gene expression and inhibit tumor growth. Through the use of advanced NP delivery systems, these therapies offer enhanced stability, targeting, and efficacy, addressing key challenges in HCC treatment. As research progresses and clinical trials continue, miRNA-based therapies have the potential to become a critical component of HCC management, offering new hope for effective and personalized cancer treatment.

### Gene silencing via siRNA nanotherapeutics

Gene silencing via RNA nanotherapeutics exhibit a cutting-edge approach for treating HCC, utilizing siRNA to target and silence specific oncogenes involved in cancer progression. These therapeutic agents are delivered through advanced NP systems that enhance siRNA stability in the bloodstream, facilitate cellular uptake, and ensure targeted delivery to HCC cells, thereby improving therapeutic outcomes. In HCC, oncogenes such as MYC, VEGF, and BCL-2 are often overexpressed, promoting uncontrolled cell proliferation, angiogenesis, and resistance to apoptosis 142, 143. By designing siRNA molecules that specifically target these oncogenes, RNA nanotherapy can effectively reduce the expression of these harmful proteins, thereby inhibiting tumor growth and progression.

Preclinical studies have demonstrated the promise of siRNA-mediated gene silencing in HCC. siRNA targeting VEGF has been shown to inhibit angiogenesis, thereby reducing the tumor's blood supply and slowing disease progression 144. Similarly, siRNA targeting MYC has significantly reduced tumor cell proliferation and induced apoptosis 145. Clinical trials are currently exploring the efficacy of siRNA therapies in HCC, with several NP-based siRNA formulations under investigation. ALN-VSP, an LNP composed of kinesin spindle protein (KSP) siRNA and VEGF siRNA in a 1:1 ratio, was well tolerated and prolonged disease stabilization in participants with nodal and extensive liver metastasis 146. These trials aim to evaluate the therapeutic effectiveness, safety, and potential side effects of these novel therapies.

Several siRNA nanotherapeutics based therapies have been designed to target specific oncogenes in HCC. For instance, siRNA targeting BCL-2, a gene that inhibits apoptosis, has shown to sensitize HCC cells to chemotherapy by promoting cell death 147. Another notable example is siRNA targeting β-catenin, a key player in the Wnt signaling pathway 148, which is crucial for HCC development and progression. By silencing β-catenin, researchers have observed reduced tumor growth and increased sensitivity to other therapeutic agents. These examples highlight the potential of siRNA therapy to disrupt critical oncogenic pathways in HCC, offering a targeted treatment approach.

**Figure 5:**
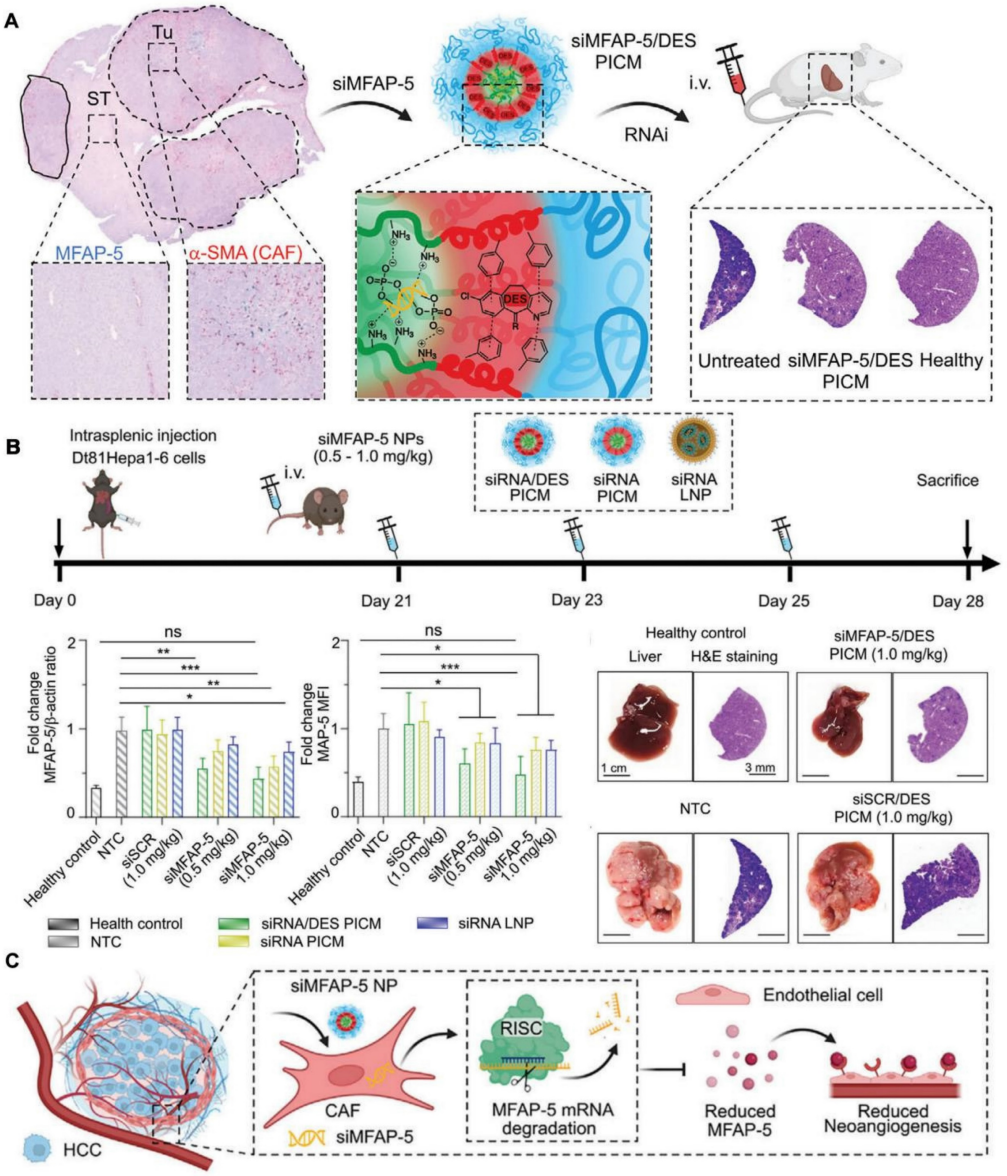
The strategy of siMFAP-5/DES PICMs in disrupting the tumor-supportive environment by targeting CAFs to treat HCC. (**A**) Schematic illustration of the siMFAP-5/DES PICM design and mechanism. The figure shows the encapsulation of siMFAP-5 in the DES PICM, which targets CAFs identified by α-SMA staining in HCC tissues. The siMFAP-5/DES PICMs are administered intravenously, leading to RNAi that specifically silences the MFAP-5 gene in CAFs within the TME. (**B**) *In vivo* treatment timeline and therapeutic outcomes in a murine HCC model. Mice injected with Dt81Hepa1-6 cells were treated with siMFAP-5 PICMs, showing a significant reduction in MFAP-5 expression in CAFs, with comparative analyses of siRNA PICMs, siRNA LNPs, and control treatments. (**C**) The mechanism is that siMFAP-5 PICMs specifically target and degrade MFAP-5 mRNA in CAFs via RISC, leading to reduced angiogenesis and tumor progression in HCC. Adapted from 149. Copyright 2024, Wiley.

Beyond directly targeting HCC cells, siRNA nanotherapy also addresses the TME, particularly cancer-associated fibroblasts (CAFs). CAFs significantly contribute to tumor progression and angiogenesis, making them crucial therapeutic targets. Recent research highlights the development of polyion complex micelles (PICMs) based on triblock polypept(o)ides, which serve as an efficient delivery system for siRNA specifically targeting microfibrillar-associated protein 5 (MFAP-5) (**Figure 5**) 149. This protein is essential in the TME, contributing to tumor growth by enhancing angiogenesis. By co-encapsulating siRNA with cationic amphiphilic drugs (CADs) like desloratadine (DES), these micelles improve endosomal escape and gene silencing efficiency, leading to significant tumor burden reduction in HCC models. This approach not only directly affects cancer cells but also disrupts the supportive TME, providing a promising strategy for HCC treatment.

The primary advantage of siRNA nanotherapeutics depend on its specificity. Unlike traditional chemotherapy, which can affect both cancerous and healthy cells, siRNA specifically targets oncogenes, minimizing off-target effects and reducing toxicity. Additionally, the use of NP delivery systems enhances siRNA stability and bioavailability, allowing for more effective delivery to tumor sites. This targeted approach also provides the potential for combination therapies, where siRNA can be used alongside other treatments to enhance overall efficacy and overcome resistance mechanisms in HCC. Recent advancements also include exploring multi-target siRNA strategies, where a single NP can deliver siRNAs targeting multiple oncogenes 150. This approach is particularly relevant in HCC, which is driven by various genetic and epigenetic alterations. By silencing multiple pathways simultaneously, researchers aim to achieve more comprehensive tumor control and reduce the likelihood of resistance development.

Gene silencing via RNA nanotherapy is an innovative and promising approach for treating HCC, leveraging the precision of siRNA to target and silence specific oncogenes involved in cancer progression. Through the use of advanced NP delivery systems, these therapies offer improved stability, targeting, and therapeutic efficacy, addressing key challenges in HCC treatment. As research continues and clinical trials progress, siRNA-based therapies have the potential to become a cornerstone of HCC management, providing patients with more effective and less toxic treatment options.

### mRNA nanotherapeutics for protein restoration in HCC

mRNA nanotherapeutics represent an innovative approach for treating HCC by directly restoring protein function through the delivery of synthetic mRNA molecules. Unlike traditional therapies that rely on small molecules or antibodies to inhibit protein activity, mRNA therapy aims to reintroduce or enhance the expression of therapeutic proteins that are missing or dysfunctional in cancer cells. These synthetic mRNAs are encapsulated in NPs, typically LNPs, which protect the mRNA from degradation, facilitate cellular uptake, and ensure effective delivery to target tissues 151. Once delivered to cells, the encapsulated mRNA is translated by the host's ribosomes into the corresponding protein, effectively restoring or enhancing the function of critical proteins that control cell growth, apoptosis, and immune responses. This approach is particularly beneficial in HCC, where certain tumor suppressor genes or regulatory proteins may be downregulated or inactivated due to genetic mutations or epigenetic modifications.

**Figure 6:**
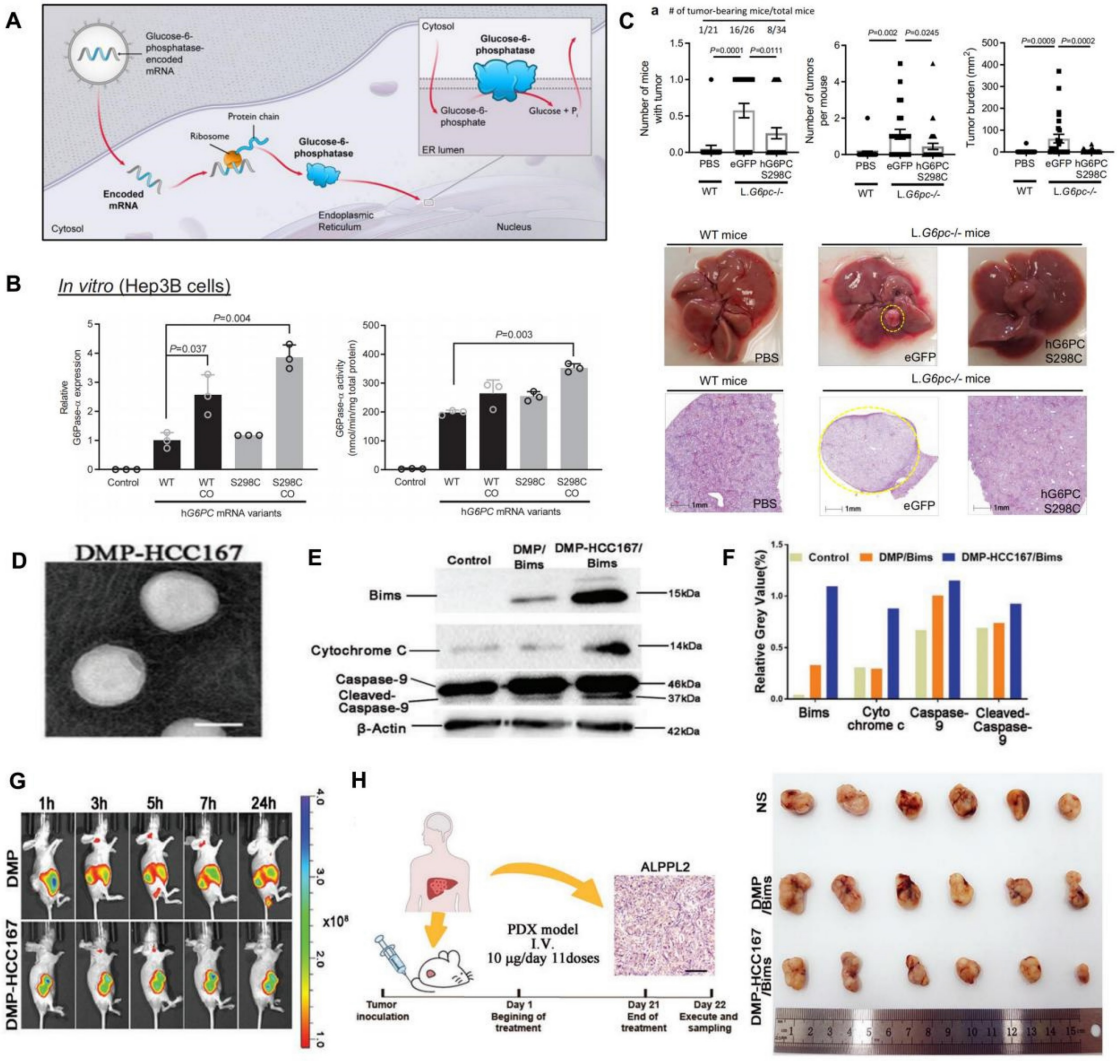
mRNA nanotherapeutics for HCC treatment. (**A**) Schematic and experimental validation of G6PC mRNA therapy for glucose-6-phosphatase deficiency, shows the mechanism of G6Pase-α mRNA delivery and expression, enabling glucose production in hepatocytes. (**B**) Validation of G6Pase-α mRNA expression and activity in Hep3B cells. (**C**) Tumor development in L.G6pc-/- mice treated with wild-type or mutant G6PC mRNA, with representative liver tumor images and histological examination in treated mice. Adapted from 155. Copyright 2021, Springer Nature. (**D**) TEM image of DMP-HCC167 NPs, designed for targeted delivery to HCC. (**E**) WB analysis shows the expression of apoptosis-related proteins in HCC cells treated with DMP-HCC167, indicating effective induction of apoptosis. (**F**) Quantification of WB. (**G**) Biodistribution study showing targeted delivery of DMP-HCC167 NPs to tumor sites in a PDX model of HCC over time, as observed via live imaging. (**H**) DMP-HCC167 NPs significantly reduced tumor size compared to control treatments in a PDX model of HCC. Adapted from 156. Copyright 2022, Wiley.

Research on mRNA nanotherapeutics for HCC has shown promising progress in preclinical models. For example, restoring the expression of tumor suppressor proteins like p53, which is often mutated or downregulated in HCC, has been a key focus. Delivering mRNA encoding functional p53 using LNPs has led to significant inhibition of tumor growth and induction of apoptosis in HCC cells in experimental models 152. Another area of focus is the delivery of mRNA-encoding cytokines and immunoregulatory proteins to enhance anti-tumor immune responses in HCC. For example, mRNA encoding IL-12 has been studied for its potential to stimulate immune cells to more effectively attack HCC tumors 153, 154. Preclinical studies have shown that IL-12 mRNA delivered via NPs can enhance immune cell infiltration into tumors, leading to improved tumor control and, in some cases, complete regression of established tumors. Therefore, IL-12 mRNA is used to stimulate the immune system to target and destroy cancer cells. Delivering IL-12 mRNA directly to tumor cells via NPs, the therapy can induce local production of IL-12 within the TME. This localized production of IL-12 enhances the immune surveillance by recruiting and activating cytotoxic T cells and NK cells to the tumor site. Additionally, metabolic dysregulation is a critical factor in the development and progression of HCC, as altered glucose and lipid metabolism provide the energy and building blocks necessary for rapid tumor growth. In particular, deficiencies in enzymes like glucose-6-phosphatase-α (G6Pase-α), which regulates glucose production in the liver, create a metabolic environment conducive to HCC. Targeting and correcting metabolic pathways offer a promising strategy for HCC treatment. As shown in **Figure 6**A-C, the study investigates the therapeutic potential of delivering G6Pase-α mRNA to restore glucose metabolism in glycogen storage disease type 1a (GSD1a), a condition characterized by G6Pase-α deficiency and a high risk of HCC 155. By delivering G6Pase-α mRNA in LNPs, the treatment aims to normalize glucose production, reduce glycogen buildup, and mitigate tumor development. The findings demonstrate effective mRNA expression, increased enzyme activity, and a reduction in liver tumor incidence in GSD1a mouse models, highlighting mRNA therapy as a potential metabolic treatment for preventing HCC.

Although mRNA delivery for HCC shows great potential, the precision of delivery to HCC cells remains a critical challenge, limiting therapeutic outcomes. To address this, a study on 1,2-dioleoyl-3-trimethylammonium-propane (DOTAP)-methoxy poly(ethylene glycol) (mPEG)-poly(ε-caprolactone) (PCL) linked HCC167 peptide (DMP-HCC167) focuses on the development of a highly specific delivery system for mRNA-based therapy targeting HCC 156. DMP-HCC167 is a phage display-derived peptide that exhibits a strong affinity for the ALPPL2 receptor, which is overexpressed in HCC cells. By conjugating this peptide to LNPs, the researchers engineered a delivery system that can precisely target and deliver therapeutic mRNA to HCC cells, thereby enhancing the therapeutic efficacy. The specificity of DMP-HCC167 for HCC cells was demonstrated through a series of experiments, showing its superior targeting capabilities compared to non-specific delivery methods (Figure 6D-H). This specific delivery is crucial as it ensures that the therapeutic mRNA is predominantly taken up by HCC cells, thereby reducing off-target effects and enhancing the treatment's overall effectiveness. The study underscores the importance of developing targeted delivery systems like DMP-HCC167 to improve the outcomes of mRNA therapies in treating HCC.

**Figure 7:**
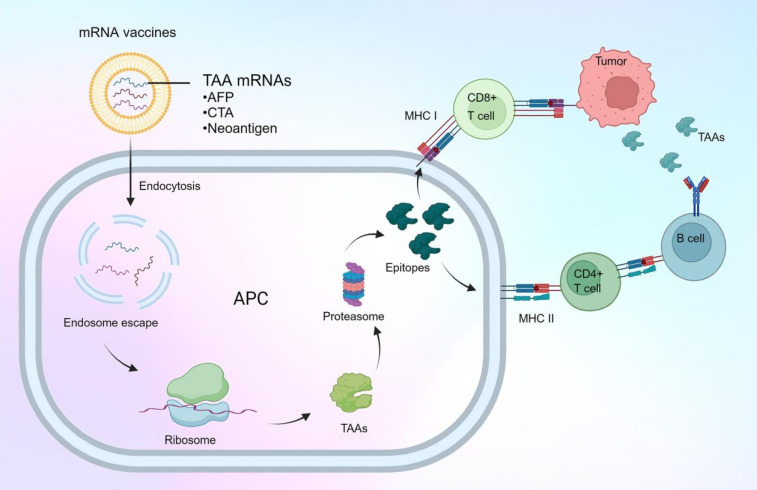
The development of mRNA HCC vaccine encoding tumor-associated antigens or neoantigens. The mRNA vaccines, which encode TAAs such as AFP, CTA, and neoantigens, are delivered into antigen-presenting cells (APCs) through endocytosis. Following endosome escape, the mRNA is translated by ribosomes into TAA proteins within the APC. These TAAs are then processed by the proteasome into smaller peptide fragments (epitopes). The epitopes are presented on the cell surface by major histocompatibility complex (MHC) molecules: MHC I presents them to CD8^+^ T cells, leading to cytotoxic T cell activation and tumor cell apoptosis, while MHC II presents them to CD4^+^ T cells, aiding in B cell activation and antibody production. This process initiates an adaptive immune response specifically targeting tumor cells expressing the TAAs. Created with BioRender.com.

Additionally, mRNA nanotherapy is making significant strides in vaccine development for HCC, representing a breakthrough in cancer immunotherapy. These vaccines are designed to stimulate the patient's immune system to recognize and attack cancer cells, providing a highly targeted approach to combating HCC 13, 157, 158. As shown in **Figure 7**, unlike traditional vaccines that often use inactivated viruses or proteins, mRNA vaccines utilize synthetic mRNA to encode tumor-associated antigens (TAAs) or neoantigens specific to a patient's tumor. To ensure effective delivery and uptake by immune cells, particularly dendritic cells, these mRNA molecules are encapsulated in LNPs. In HCC, mRNA vaccine development has focused on targeting specific TAAs such as alpha-fetoprotein (AFP) and GPC3 159, which are often overexpressed in liver cancer cells. By targeting these antigens, mRNA vaccines can help direct the immune system to selectively attack HCC cells while sparing normal liver tissue.

In addition to targeting known TAAs, researchers are also exploring the use of personalized mRNA vaccines targeting neoantigens, which are generated by tumor-specific mutations. This approach is particularly promising in HCC, which often exhibits high genetic heterogeneity. By sequencing a patient's tumor and identifying neoantigens, researchers can create personalized mRNA vaccines that target the unique mutation profile of the tumor, potentially leading to more effective and durable immune responses.

mRNA vaccines for HCC treatment offer several notable advantages 160. First, mRNA vaccines are non-infectious and non-integrative, eliminating the risk of insertional mutagenesis and other genetic alterations. They are also highly adaptable, allowing for rapid design and production in response to emerging tumor antigens or changes in a patient's tumor profile. This adaptability is particularly valuable in personalized medicine, where treatments can be tailored to the specific needs of the patient.

mRNA nanotherapeutics provide a promising approach for HCC treatment through protein restoration and vaccine development. By encapsulating mRNA in LNPs, therapeutic proteins such as tumor suppressors are directly delivered to HCC cells, helping to inhibit tumor growth and enhance treatment efficacy. As vaccines, mRNA nanotherapeutics encode tumor-associated antigens, stimulating the immune system to target and destroy cancer cells. These strategies have shown significant potential in preclinical and early clinical trials, offering new targeted and adaptable treatment options for HCC.

### saRNA nanotherapeutics for HCC via protein expression enhancement

saRNA nanotherapeutics represent a novel approach to HCC treatment by enhancing the expression of beneficial genes rather than silencing them like siRNA. This mechanism, known as RNA activation (RNAa), differs from the gene-silencing effect of siRNA, allowing for the reactivation of tumor suppressor genes or other therapeutic genes that are downregulated in cancer cells. When delivered using advanced NP systems such as LNPs or PNPs, saRNAs can be effectively transported to target cells, where they bind to the promoter regions of specific genes 161. This binding recruits transcription factors, leading to increased transcription and expression of the target gene. In HCC, the downregulation of tumor suppressors and other regulatory genes is common, and saRNA therapy offers the potential to restore normal cellular function and inhibit tumor progression 162.

**Figure 8:**
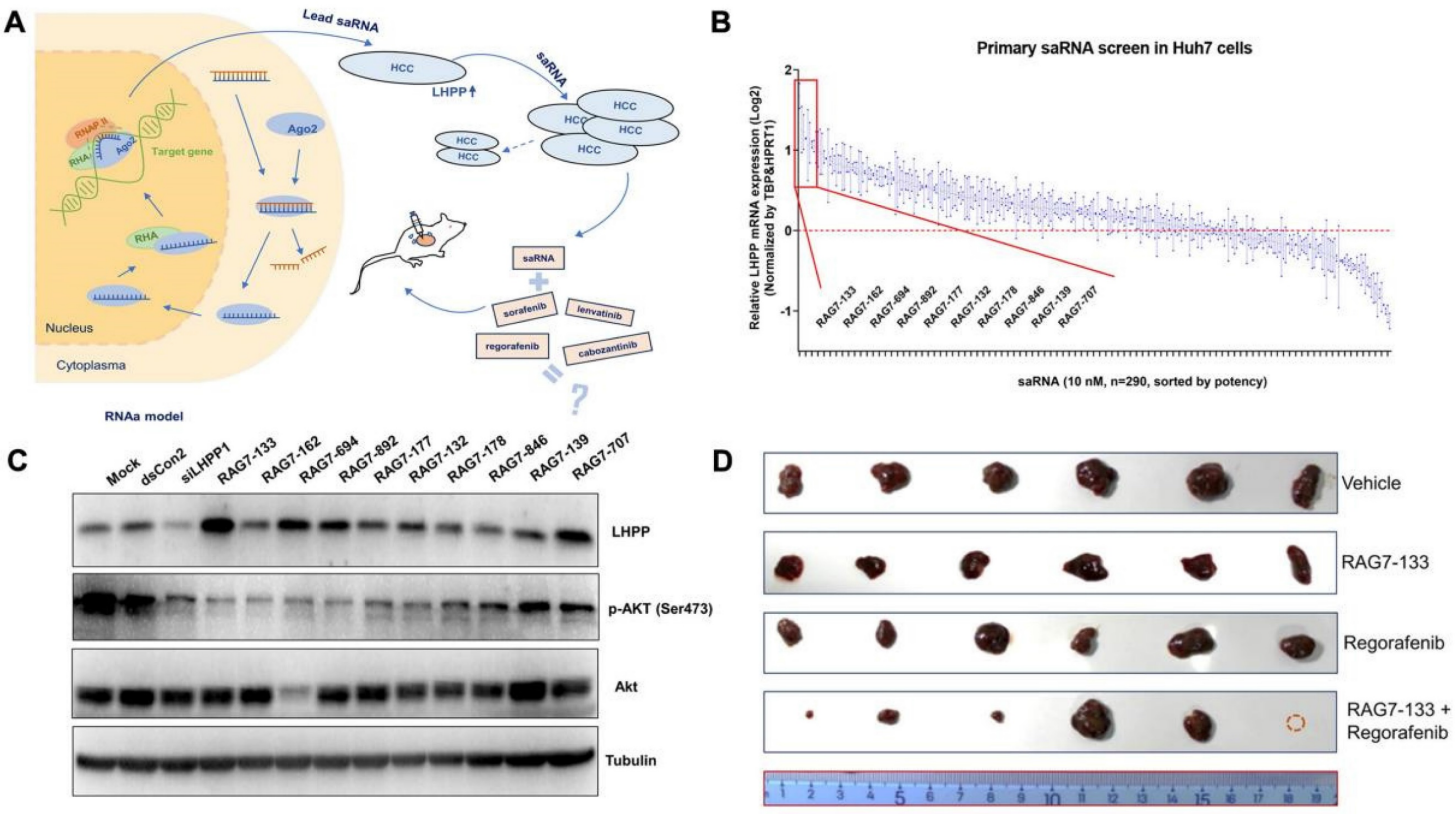
The therapeutic potential of LHPP-saRNA in suppressing HCC treatment. (**A**) Schematic illustration of RNA activation mechanism to upregulate the tumor suppressor LHPP using saRNA in HCC cells. The saRNA forms a complex with Ago2, facilitating targeted LHPP expression and potentially reducing HCC progression. (**B**) Screening results of saRNA candidates in Huh7 cells, showing relative LHPP mRNA expression for each saRNA tested. (**C**) Western blot analysis of LHPP, p-AKT, and Akt levels in HCC cells treated with lead saRNAs, demonstrating successful LHPP upregulation and AKT pathway inhibition. (**D**) Representative images of tumor growth in mouse xenograft models treated with vehicle, RAG7-133, regorafenib, and the combination, showing enhanced anti-tumor effects with RAG7-133 and regorafenib co-treatment. Adapted from 168. Copyright 2024, Public Library of Science.

The development of saRNA nanotherapeutics for HCC is an emerging field, with significant progress made in preclinical studies. One key target for saRNA therapy in HCC is the E-cadherin gene, which is often downregulated in HCC, leading to increased invasiveness and metastasis 163. Research also investigated the role of CEBP/α, a tumor suppressor transcription factor, in the inhibition of HCC metastasis 164. The study utilizes saRNAs to enhance the expression of CEBP/α *in vitro* and *in vivo*. The research demonstrates that CEBP/α-saRNA transfection inhibits the migration and invasion of hepatoma cells by suppressing the EMT process 165. The study highlights the significant downregulation of key EMT markers, such as N-Cadherin, Slug, and Vimentin, alongside the upregulation of E-Cadherin, indicating a reversion to a less invasive epithelial phenotype. Another important target is the p21 gene, a cyclin-dependent kinase inhibitor that plays a crucial role in cell cycle regulation and apoptosis 166. The expression of p21 is often suppressed in HCC, leading to uncontrolled cell proliferation. Preclinical studies have demonstrated that saRNA targeting the p21 promoter can enhance its expression, leading to cell cycle arrest and increased sensitivity of HCC cells to chemotherapy 167. LHPP, a recently identified tumor suppressor, has shown promise as a target for therapeutic intervention in HCC. As shown in **Figure 8**, researchers utilized a systematic screening process to identify effective saRNAs that can upregulate the tumor suppressor gene LHPP in HCC cells 168. The screening involved designing and synthesizing a library of 290 saRNA candidates, each specifically targeting different regions of the LHPP promoter. One lead saRNA, RAG7-133, demonstrated significant inhibition of tumor growth and enhanced therapeutic efficacy in combination with regorafenib *in vitro* and *in vivo*, suggesting that saRNA-mediated LHPP activation could be a novel approach for HCC treatment.

Particularly, saRNA nanotherapeutics are valuable in restoring the function of tumor suppressors and other regulatory genes critical for controlling cell proliferation, apoptosis, and metastasis. Additionally, saRNA therapy can be designed to target multiple genes simultaneously, providing a broader impact on disease. The use of NP delivery systems further enhances the effectiveness of saRNA by protecting the RNA from degradation and ensuring its efficient delivery to target cells. This targeted delivery minimizes off-target effects and reduces the likelihood of systemic side effects, making saRNA nanotherapy a safer alternative to traditional treatments. Moreover, the transient nature of saRNA expression reduces the risk of long-term adverse effects, offering a balance between efficacy and safety.

### RNA nanotherapeutics in combination therapy for HCC

When RNA nanotherapeutics is combined with other treatment modalities, it offers a powerful approach to combination therapy for HCC. This strategy leverages the unique capabilities of RNA molecules such as siRNA, miRNA, and mRNA to target specific oncogenes and regulatory pathways, while concurrently employing traditional or novel cancer treatments such as chemotherapy, targeted therapy, immunotherapy, or radiation therapy 169-171. By combining these approaches, overall therapeutic efficacy is enhanced, and the limitations of individual treatments, such as resistance or toxicity, can be mitigated.

The mechanism of action of RNA nanotherapeutics in combination therapy often involves silencing or modulating genes that promote cancer progression, drug resistance, or immune evasion. For example, siRNA can be used to silence genes responsible for chemotherapy resistance, making cancer cells more susceptible to chemotherapeutic agents 172. Meanwhile, mRNA can be used to restore the expression of tumor suppressor genes or enhance immune responses against cancer cells 173. The use of NP delivery ensures that RNA molecules are effectively transported to the tumor site, protected from degradation, and released in a controlled manner, maximizing therapeutic benefit.

Research on RNA nanotherapeutics in combination with other therapies is an active field, with many promising advances in both preclinical and clinical settings. A notable example is the use of siRNA targeting BCL-2, an anti-apoptotic gene often overexpressed in HCC that contributes to chemotherapy resistance 174. In preclinical studies, siRNA targeting BCL-2 combined with Sorafenib has shown enhanced cancer cell death and tumor regression, outperforming either treatment alone. This combination approach is being explored in early clinical trials to assess its safety and efficacy in HCC patients.

**Figure 9:**
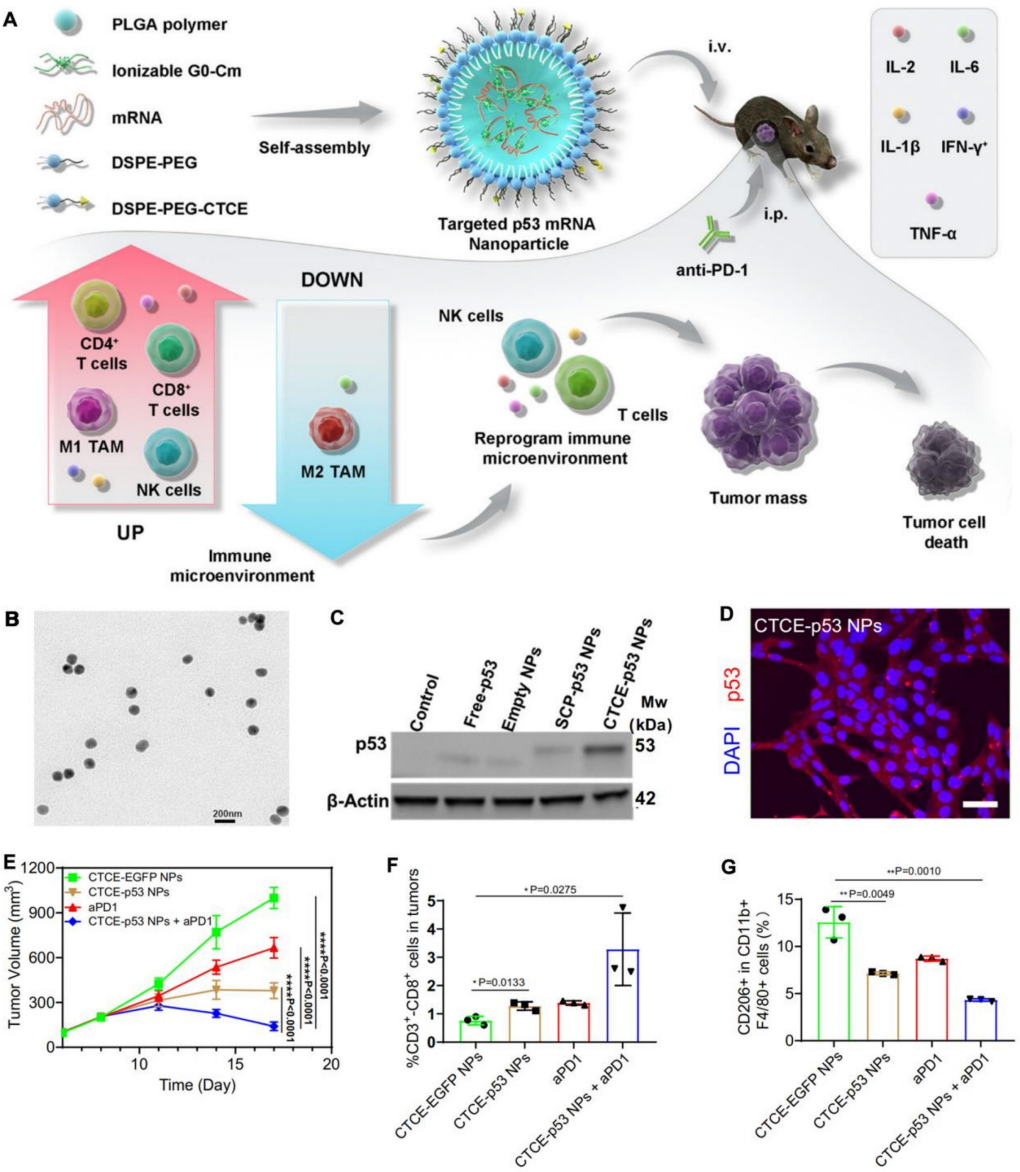
CTCE-p53 NPs for enhancing anti-tumor immune response in HCC. (**A**) Schematic illustration showing the composition and mechanism of CTCE-p53 NPs in reprogramming the immune microenvironment and enhancing anti-tumor immunity when combined with anti-PD-1 therapy. These NPs upregulate CD4^+^ and CD8^+^ T cells, M1 TAMs, and NK cells while downregulating M2 TAMs. (**B**) TEM image showing the morphology of the CTCE-p53 NPs (scale bar: 200 nm). (**C**) Western blot analysis confirms the expression of p53 protein in treated HCC cells. (**D**) Immunofluorescence image of p53 protein expression in p53-knockout RIL-175 cells treated with CTCE-p53 NPs (scale bar: 50 μm). (**E**) Tumor growth curves in p53-null RIL-175 tumor-bearing mice treated with different formulations. (**F**) Quantification of CD3^+^CD8^+^ T cells infiltrating tumors, and (**G**) CD206^+^ M2-like TAMs in tumors, demonstrating enhanced immune response and reduced immunosuppressive TAMs in mice treated with CTCE-p53 NPs combined with anti-PD-1 therapy. Adapted from 27. Copyright 2022, Springer Nature.

Another focus of research is the combination of RNA nanotherapeutics with immune checkpoint inhibitors such as anti-PD-1/PD-L1 antibodies. In this approach, a study utilizes a CXCR4 targeted p53 mRNA-loaded nanoparticle, named CTCE-p53 NPs, to restore the function of the tumor suppressor p53 in HCC cells (**Figure 9**) 27. These NPs are engineered with PLGA polymer, ionizable lipids, and CTCE-PEG, which specifically targets the TME. The CTCE-p53 NPs not only induce the re-expression of p53, leading to apoptosis in p53-null cancer cells, but also reprogram the immune microenvironment. This reprogramming increases the infiltration of CD8^+^ T cells and natural killer (NK) cells while reducing the population of immunosuppressive M2-like macrophages, thus enhancing the overall anti-tumor immune response. When combined with anti-PD-1 therapy, the CTCE-p53 NPs significantly amplify the therapeutic effects by overcoming the immune evasion mechanisms typically employed by tumors. This dual strategy effectively suppresses tumor growth more than either treatment alone, highlighting the innovative synergy between RNA nanotherapeutics based nanotherapy and immune checkpoint blockade. The results from this study suggest that combining p53 mRNA nanotherapy with PD-1 inhibition could offer a powerful, complementary approach to treating HCC and potentially other cancers characterized by p53 dysfunction. This combination approach has shown promise in preclinical models, leading to improved tumor control and extended survival in HCC mouse models.

Beyond chemotherapy and immunotherapy, RNA nanotherapeutics is also being combined with targeted therapies such as tyrosine kinase inhibitors (TKIs). For example, siRNA targeting MYC or β-catenin, combined with sorafenib (a commonly used TKI in HCC), has demonstrated synergistic effects in reducing tumor growth and overcoming resistance to Sorafenib 175. These findings highlight the potential of RNA nanotherapeutics to enhance the effectiveness of existing targeted therapies and provide new treatment options for HCC patients who do not respond to standard treatments.

Combining RNA nanotherapeutics with other treatment modalities offers several advantages 176. First, it allows for a multifaceted attack on tumors, targeting different aspects of cancer biology simultaneously. This approach helps overcome the limitations of single therapies, such as drug resistance or incomplete tumor eradication. Second, the use of NPs for RNA delivery enhances the stability and bioavailability of RNA molecules, ensuring that they reach the tumor site in sufficient quantities to exert therapeutic effects.

Additionally, combining RNA therapy with other treatments can reduce the required dose of chemotherapy or other drugs, minimizing toxicity and improving the overall safety of the treatment regimen.

RNA nanotherapeutics in combination with other treatments represents a promising strategy for HCC combination therapy. Preclinical and early clinical research has demonstrated the potential of these combination approaches to improve tumor control, overcome resistance, and reduce treatment-related toxicity 177. As research continues, RNA-based combination therapies are likely to play an increasingly important role in HCC management, offering new hope for more effective and personalized treatment options.

In summary, RNA nanotherapeutics represent a cutting-edge approach for HCC treatment, utilizing nanocarriers to deliver various RNA molecules, such as siRNA, miRNA, mRNA, and saRNA, directly to tumor cells. These therapies are designed to precisely target genetic pathways involved in cancer progression, offering enhanced stability, cellular uptake, and specificity compared to traditional methods. The following **Table 2** provides an overview of key examples of RNA nanotherapeutics in HCC treatment, highlighting their mechanisms of action, delivery systems, and therapeutic outcomes.

## Outlook

### Opportunities and challenges of RNA nanotherapeutics for HCC

While still in various stages of research and clinical development, RNA nanotherapies hold immense potential to provide more effective, personalized, and less toxic treatment options for HCC 179. However, to fully realize the potential of RNA nanotherapeutics for HCC, several critical challenges in clinical translation must be addressed.

#### How to achieve efficient and specific delivery of RNA nanotherapeutics

A primary challenge is the efficient and specific delivery of RNA therapies to tumor sites, which requires navigation through a complex TME characterized by a dense extracellular matrix, abnormal vasculature, and immune barriers 180. Precise targeting of HCC cells is crucial to maximizing therapeutic efficacy while minimizing off-target effects on healthy tissues. RNA molecules are inherently unstable and prone to degradation by nucleases in the bloodstream, and even when encapsulated in NPs, maintaining their stability until they reach target cells remains a significant challenge 181. Strategies to enhance stability include chemical modifications of RNA or the development of more robust NP formulations, areas that are under active investigation.

Developing targeted delivery systems that utilize ligands or antibodies on NP surfaces to specifically bind to receptors overexpressed on HCC cells, such as ASGPR, GPC3, or EGFR, is essential. Additionally, leveraging the enhanced permeability and retention effect in tumors can improve NP accumulation at the tumor site. Advances in biomimetic delivery systems, such as exosomes that naturally target specific tissues, may also enhance targeting specificity 182. Enhancing the chemical stability of RNA molecules through modifications such as 2'-O-methyl or 2'-fluoro substitutions can make them more resistant to nuclease degradation 183. Optimizing NP formulations, including protective polymers or the use of nanocages, can further protect RNA from degradation. Encapsulating RNA in NPs with pH-sensitive or enzyme-responsive release mechanisms ensures that RNA remains stable until it reaches the TME.

#### Biosafety concerns of the nanocarriers

While NPs offer protection against RNA degradation, they can also provoke immune responses 184, particularly with synthetic delivery systems like LNPs. These immune reactions may activate the innate immune system, resulting in the rapid clearance of the nanoparticles, which compromises therapeutic effectiveness and can lead to adverse effects such as inflammation or allergic reactions. The risk of immune activation is especially pronounced with repeated dosing, which can further amplify immune recognition and result in diminished therapeutic efficacy over time.

To address these challenges, balancing the immunogenicity of delivery systems with their efficacy has become a key focus in nanomedicine development. Refining the composition of LNPs to minimize immunogenicity, such as using ionizable lipids less likely to trigger immune responses, is also crucial 69. Integrating polyethylene glycol (PEG) coating can reduce the risk of immune system recognition, though care must be taken to avoid the “PEGylation syndrome”, where repeated dosing can lead to immune reactions against PEG.

An emerging solution is the use of biomimetic carriers, such as exosomes or cell membrane-coated nanoparticles, which inherently have lower immunogenicity compared to synthetic nanoparticles 185. These biomimetic vectors can evade immune detection more effectively due to their natural origin, making them well-suited for repeated administration.

Additionally, surface modifications such as incorporating self-peptides or anti-inflammatory molecules can further reduce immune recognition.

Moreover, the integration of machine learning into the discovery and development of LNPs significantly enhances the precision and efficiency of mRNA delivery systems 186, 187. Machine learning offers several advantages, such as the ability to process vast datasets and identify optimal ionizable lipids, which are critical for mRNA delivery. By combining machine learning with advanced combinatorial chemistry, researchers can rapidly explore extensive libraries of potential lipid candidates, accelerating the identification of lipids that enhance transfection efficacy and stability. This approach not only expedites the development of effective LNPs but also allows for the fine-tuning of LNP formulations to address specific therapeutic needs.

#### How to design personalized and adaptive approaches for overcoming HCC heterogeneity

HCC is a highly heterogeneous disease, with significant genetic and epigenetic variations between tumors and even within different regions of the same tumor 188. This intratumoral and intertumoral heterogeneity significantly complicates treatment efforts, as these variations can influence the tumor's response to therapies and promote resistance. The inherent diversity of HCC results in the emergence of resistant cancer cell subpopulations, even in the presence of otherwise effective treatment. Consequently, overcoming this heterogeneity is one of the most pressing challenges in advancing effective HCC therapies. To meet this challenge, personalized and adaptive treatment approaches are essential.

One promising approach is the discovery of new HCC-related genes through clinical analysis, which can provide a basis for developing personalized RNA-based therapeutics. Techniques such as next-generation sequencing (NGS), single-cell RNA sequencing (scRNA-seq), and whole-genome methylation analysis can be employed to identify critical genetic and epigenetic alterations in individual HCC patients 189. By examining patient-derived tumor samples, novel oncogenes and tumor suppressor genes that contribute to disease progression can be identified. This knowledge allows for the precise design of RNA therapeutics tailored to modulate specific gene expression patterns. The use of bioinformatics tools, such as machine learning algorithms, can further aid in the discovery and validation of these gene targets 190. Machine learning approaches can identify gene expression patterns that correlate with poor prognosis or treatment resistance, thus helping to prioritize targets for RNA-based intervention. This process of correlating clinical data with specific genetic mutations or expression levels can guide the design of co-related RNA therapeutics, maximizing therapeutic efficacy while minimizing off-target effects.

Therefore, by analyzing the genetic and molecular characteristics of each patient's tumor, personalized medicine approaches can tailor RNA-based therapies to individual needs 191. This can include using siRNA or miRNA to target specific oncogenic pathways, or mRNA or saRNA to enhance tumor suppressor activity, thereby providing more precise and effective treatment while reducing off-target effects. Combination therapies targeting multiple pathways simultaneously, such as using siRNA in combination with chemotherapy or immune checkpoint inhibitors, can help overcome resistance and address tumor heterogeneity. The development of adaptive treatment regimens that evolve with changes in tumor biology can further improve long-term efficacy 192, ensuring that therapies remain effective even as the disease progresses. Integrating these innovations with machine learning-enhanced LNP discovery can lead to more effective and personalized RNA-based nanotherapies, optimizing therapeutic outcomes for patients.

## Conclusion

RNA nanotherapeutics represent an innovative approach in HCC treatment, harnessing the precision of RNA molecules such as siRNA, miRNA, mRNA, and saRNA, in combination with advanced NP delivery systems to specifically target cancer cells. These therapies offer the potential to silence oncogenes, restore tumor suppressor functions, and modulate immune responses, providing a highly targeted approach that may overcome the high toxicity, limited specificity, and drug resistance associated with conventional treatments. By personalizing therapy to address specific genetic and molecular alterations in individual tumors, RNA nanotherapeutics can effectively tackle the heterogeneity inherent in HCC, enhancing treatment efficacy while minimizing off-target effects.

To achieve successful clinical translation of RNA nanotherapeutics, a multifaceted strategy is required. This includes conducting thorough scientific research to optimize delivery systems and ensure safety, collaborating with regulatory agencies to establish clear approval pathways, developing scalable manufacturing processes for widespread accessibility, strategically integrating these therapies into clinical practice, potentially in combination with existing treatments. By focusing on these critical areas, the medical community can advance RNA nanotherapeutics toward becoming a viable and impactful option for patients battling HCC, ultimately improving outcomes and offering new hope in the management of this challenging disease.

In summary, recognizing the burgeoning trend towards developing more precise and functional nanocarriers, we explore how these cutting-edge strategies can enhance tumor specificity and RNA accumulation, thereby facilitating further clinical translation and advancing cancer therapy. As strides are made in nanotechnology and our understanding of cancer deepens, we believe that these RNA nanotherapeutics will not only overcome the limitations of conventional treatments but also set a new benchmark in the next generation of nano-anticancer agents. Ultimately, this review aims to highlight the potential of RNA nanotherapeutics to become a paragon in personalized medicine for HCC, offering hope for more effective and less toxic treatment options.

## Figures and Tables

**Table 1:** Comparison of different types of vectors for RNA delivery

Vector type	Examples	Advantages	Challenges	Applications
Viral Vectors	AAV, lentivirus, adenovirus	High transduction efficiency, sustained gene expression	Immunogenicity, risk of insertional mutagenesis, complex and costly production	Gene therapy (e.g., Luxturna™, Zolgensma™)
Bioinspired Vectors	VLPs, Nanocages, Exosomes, EVs	High biocompatibility, reduced immunogenicity, natural targeting mechanisms	Complex production and purification, variability in composition	Cancer therapy, targeted RNA delivery
Lipid Nanoparticles	Commonly used for mRNA and siRNA delivery	High biocompatibility, enhances cellular uptake, protects RNA from degradation	Potential immunogenicity, requires sophisticated manufacturing	mRNA vaccines (e.g., COVID-19 vaccines), gene therapy
Polymer Nanoparticles	PLGA, PEI-based NPs	Biocompatible, customizable surface chemistry for targeted delivery	Potential cytotoxicity, variability in synthesis leading to inconsistent results	siRNA delivery, cancer therapy
Other vectors	MOFs, QDs, Mesoporous silica NPs	high surface area, tunable porosity, pH-responsive release	Biodegradability concerns, potential metal ion toxicity	Gene silencing in cancer models

**Table 2:** List of representative RNA nanotherapeutics applications in HCC treatment

RNA type	Vector type	Combination Therapy	Target Receptor	Outcomes	Trail Phase	Ref
miR-26a mimics	Exsome	N/A	N/A	Inhibition of cell proliferation, induced cell cycle arrest	Preclinical	139
miR-34a mimics	LNPs	N/A	N/A	Targeting MYC and BCL-2, reduced tumor growth, increased apoptosis	Early Clinical Trials	137
miR-122 mimics	LNPs	N/A	N/A	Restoration of miR-122 levels, tumor growth inhibition	Preclinical	135
VEGF siRNA	PNPs	5-FU	VEGFR	Inhibition of angiogenesis, reduced tumor blood supply	Preclinical	144
MYC siRNA	PNPs	Sorafenib	asialoglycoprotein receptor	Reduced tumor cell proliferation, induced apoptosis	Preclinical	145
BCL-2 siRNA	PNPs	N/A	N/A	Enhanced cancer cell death, tumor regression	Preclinical	147
β-catenin siRNA	LNPs	Sorafenib	β-catenin receptor	Reduced tumor growth, overcoming Sorafenib resistance	Preclinical	148
ALN-VSP	LNPs	N/A	N/A	Well-tolerated and prolonged disease stabilization in participants	Phase 1	146
MFAP-5 siRNA	PNPs	Desloratadine	N/A	Significant tumor burden reduction, inhibited angiogenesis	Preclinical	149
IL-12 mRNA	LNPs	N/A	Not specified	Immune modulation, suppression of tumorigenesis	Preclinical	153
p53 mRNA	PNPs	Anti-PD-1	CXCR4 receptor	Restoration of p53 expression, enhanced immune response	Preclinical	27
OX40L mRNA	LNPs	N/A	N/A	Inhibits tumor growth, increases CD4^+^ and CD8^+^ T cell populations, promotes immune infiltration	Preclinical	178
Bims mRNA	ALPPL2-Binding Peptide	N/A	ALPPL2	Enhanced targeted delivery, significant tumor growth inhibition	Preclinical	156
CEBPA saRNA	SMARTICLES	TKIs	N/A	Upregulation of CEBPA, tumor shrinkage, improved liver function	Phase I	63
CEBPA saRNA	Lipopolyplex	N/A	N/A	Gene activation, tumor growth inhibition in colorectal cancer model	Preclinical	165
p21 saRNA	Lipopolyplex	N/A	N/A	Cell cycle arrest and inhibition of invasion and migration	Preclinical	167

## Abbreviations

HCC: Hepatocellular carcinoma
LNP: lipid nanoparticle
PNP: polymer nanoparticle
miRNA: micro RNA
siRNA: small interfering RNA
mRNA: message RNA
saRNA: small activating RNA
RNA interference: RNAi
TME: tumor microenvironment
EV: Extracellular vesicle
Virus-like particle: VLP
adeno-associated viruses: AAV
LSEC: liver sinusoidal endothelial cells
extracellular matrix: ECM
Myeloid-derived suppressor cells: MDSCs
Cancer-associated fibroblasts: CAFs
Epithelial-mesenchymal transition: EMT
asialoglycoprotein receptor: ASGPR
N-acetylgalactosamine: GalNAc
Glypican-3: GPC3
RNA-induced silencing complex: RISC
Vascular endothelial growth factor: VEGF
Polyion complex micelles: PICMs
Microfibrillar-associated protein 5: MFAP-5
Tumor-associated antigens: TAAs
Major histocompatibility complex: MHC
Tekmira-Pololike Kinase 1: TKM-PLK
epidermal growth factor receptor: EGFR
poly(lactic-co-glycolic acid): PLGA
polyethylenimine: PEI
matrix metalloproteinase: MMP
glucose-6-phosphatase-α: G6Pase-α
